# Design, synthesis and *in vitro* biological evaluation of quinazolinone derivatives as EGFR inhibitors for antitumor treatment

**DOI:** 10.1080/14756366.2020.1715389

**Published:** 2020-01-22

**Authors:** Yi Le, Yiyuan Gan, Yihong Fu, Jiamin Liu, Wen Li, Xue Zou, Zhixu Zhou, Zhenchao Wang, Guiping Ouyang, Longjia Yan

**Affiliations:** a State Key Laboratory Breeding Base of Green Pesticide and Agricultural Bioengineering, Key Laboratory of Green Pesticide and Agricultural Bioengineering, Ministry of Education, State-Local Joint Laboratory for Comprehensive Utilization of Biomass, Center for Research and Development of Fine Chemicals, Guizhou University, Guiyang, China; b School of Pharmaceutical Sciences, Guizhou University, Guiyang, China; c Guizhou Engineering Laboratory for Synthetic Drugs, Guiyang, China; d Clinical Research Center, Affiliated Hospital of Guizhou Medical University, Guiyang, China

**Keywords:** Quinazolinone, EGFR, tyrosine kinase inhibitor, antitumor, molecular docking

## Abstract

In this paper, a series of novel 3-methyl-quinazolinone derivatives was designed, synthesised and evaluated for antitumor activity *in vitro* on wild type epidermal growth factor receptor tyrosine kinase (EGFR^wt^-TK) and three human cancer cell lines including A549, PC-3, and SMMC-7721. The results displayed that some of the compounds had good activities, especially 2-{4-[(3-Fluoro-phenylimino)-methyl]-phenoxymethyl}-3-methyl-3H-quinazolin-4-one (**5 g**), 2-{4-[(3,4-Difluoro-phenylimino)-methyl]-phenoxymethyl}-3-methyl-3H-quinazolin-4-one (**5k**) and 2-{4-[(3,5-Difluoro-phenylimino)-methyl]-phenoxymethyl}-3-methyl-3H-quinazolin-4-one (**5 l**) showed high antitumor activities against three cancer cell lines. Moreover, compound **5k** could induce late apoptosis of A549 cells at high concentrations and arrest cell cycle of A549 cells in the G2/M phase at tested concentrations. Also, compound **5k** could inhibit the EGFR^wt^-TK with IC_50_ value of 10 nM. Molecular docking data indicates that the compound **5k** may exert inhibitory activity by forming stable hydrogen bonds with the R817, T830 amino acid residues and cation-Π interaction with the K72 residue of EGFR^wt^-TK.

## 1. Introduction

**Figure 1.**
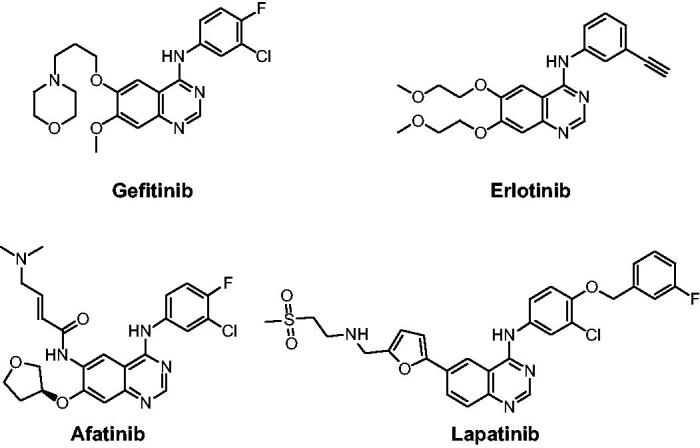
EGFR inhibitors in drugs.

Epidermal growth factor receptor (EGFR) tyrosine kinase (TK) plays an indispensable role in cancer cell proliferation, survival, adhesion, migration and differentiation. Overexpression and mutation of EGFR have been associated with a variety of cancers^1^^,^^2^. Development of EGFR inhibitors has become a main focus in antitumor drug campaigns. At present, several drugs such as Gefitinib, Erlotinib, Afatinib, and Lapatinib, have been approved by the Food and Drug Administration (FDA) for clinical use as EGFR inhibitors (Figure 1)^3^^,^^4^. However, the emergence of acquired point mutations has weakened their therapeutic efficacy, leading to drug resistance and toxicity burden^5^. Consequently, it is an urgent goal to discover new structural EGFR inhibitors for cancer therapy^6^.

**Figure 2.**
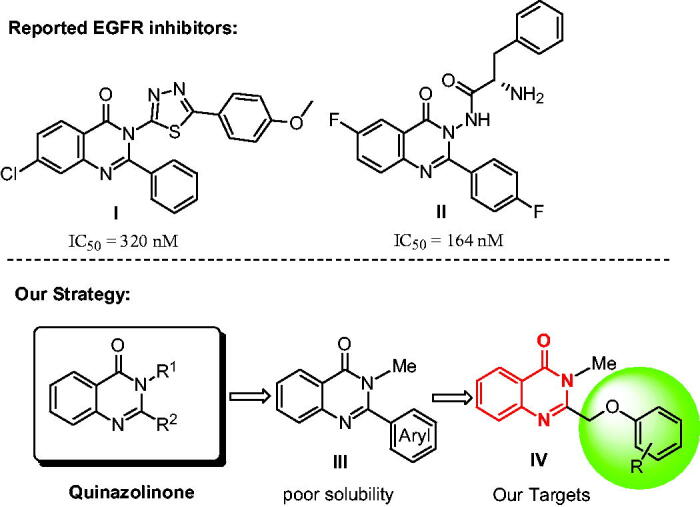
Structures of reported compounds and design of novel quinazolinones.

Quinazolinone is a heterocyclic scaffold exhibiting a broad range of biological activities^7^, such as antitumor, antimicrobial, anti-inflammatory, anticonvulsant, antiviral, antidiabetic and insecticidal activities^8^^,^^9^. The derivatives of quinazolinone can exert anti-tumour activity by inhibiting EGFR-TK, cyclin dependent kinase 4 (CDK4)^10^, tubulin polymerization^11^, HER2^12^, VEGFR^13^ and c-Met^14^. There are many reports of 3-position substituted quinazoline derivatives as EGFR inhibitors, such as compound **I**^15^ and **II**^16^ in Figure 2. However, the works as EGFR inhibitors of quinazoline in 2-position were few reported in literature^17^.

Based on the bioactivities of quinazolinone, our laboratory is interested in the development of the novel EGFR inhibitors. Initially, we designed a new series of 2-substituted quinazolinone derivatives for EGFR-TK inhibitors, compound **III** in Figure 2. However, in the previous studies, we found that the 2-arylquinazolinones had poor solubility. Therefore, we used the ethyl ether group as the linker for increasing the soluble activity, and then we designed a novel 3-methylquinazolinones compound **IV** as shown in Figure 2. In this paper, we synthesised the new compounds and tested the activities against EGFR^wt^-TK with ELISA assay. Meanwhile, *in vitro* cytotoxicity of compound **IV** against human prostate cancer cells (PC-3), human lung cancer cells (A549), human liver cancer cells (SMMC-7721), and normal rat kidney cell (NRK-52E) were evaluated by MTT method.

## 2. Experimental section

NMR spectroscopic data were recorded with Bruker 400 MHz NMR spectrometer (400 MHz for ^1^H and 100 MHz for ^13 ^C) in DMSO-*d*_6_ or CDCl_3_ solution, with TMS serving as the internal standard. Mass spectra were recorded with TSQ 8000 high-resolution mass spectrometer (HRMS, Thermo Fisher Scientific Co., Ltd.). Melting points were determined with X-4X digital display micro melting point analyser (uncorrected, Shanghai Microelectronics Technology Co., Ltd.).

### 2.1. Synthesis

**Scheme 1.**
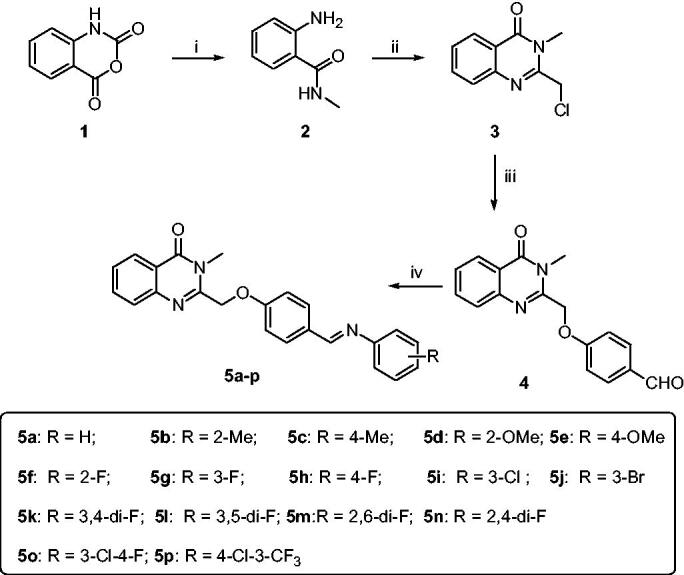
Synthetic route of target compounds **5a-5r**. Reagents and conditions: (a) CH_3_NH_2_, DMF, 50 °C; (b) 2-chloroacetyl chloride, AcOH, reflux; (c) 4-hydroxybenzaldehyde, K_2_CO_3_, KI, DMF, rt; (d) R-aniline, AcOH, toluene, reflux.

The synthetic route of the target compounds is shown in Scheme 1. The synthetic methods of intermediates 2–5 followed the previous procedures, and the detail process is described as follows.

#### 2.1.1. Synthesis of 2-amino-N-methylbenzamide (2)

The solution of isatoic anhydride (1.00 g, 6.13 mmol), 30% MeNH_2_ aqueous solution (0.96 ml, 9.20 mmol) and THF (10 ml) was stirred at room temperature for 1 h. Then ethyl acetate (20 ml) and saturated brine (10 ml) were added. The organic layer was separated and washed with water, dried with anhydrous Na_2_SO_4_, filtered and concentrated *in vacuo* to afford compound **2**. 0.45 g white solid; 49% yield; ^1^H NMR (400 MHz, CDCl_3_) δ 7.26 (d, *J* = 8.0 Hz, 1H), 7.12 (t, *J* = 7.2 Hz, 1H), 6.61 (d, *J* = 8.0 Hz, 1H), 6.54 (t, *J* = 7.2 Hz, 1H), 6.51 (br, 1H), 5.38 (s, 2H), 2.84 (dd, *J* = 4.8, 0.8 Hz, 3H). Spectral properties were in accordance with the literature^18^.

#### 2.1.2. Synthesis of 2-(chloromethyl)-3-methylquinazolin-4(3H)-one (3)

The solution of compound **2** (1.00 g, 6.67 mmol), 2-chloroacetyl chloride (2.26 g, 20.01 mmol) and glacial acetic acid (50 ml) was heated to reflux for 10 h. Then, the mixture was concentrated *in vacuo*, neutralised with aqueous Na_2_CO_4_. After filtration, the residue was recrystallized with isopropyl alcohol to afford compound **3**. 0.68 g white solid; 49% yield; ^1^H NMR (400 MHz, CDCl_3_) δ 8.29 (d, *J* = 8.0 Hz, 1H), 7.80–7.75 (m, 1H), 7.68 – 7.69 (m, 1H), 7.50–7.54 (m, 1H), 4.65 (s, 2H), 3.76 (s, 3H). Spectral properties were in accordance with the literature^19^.

#### 2.1.3. Synthesis of 4-((3-methyl-4-oxo-3,4-dihydroquinazolin-2-yl)methoxy)benzaldehyde (4)

To a 50 ml-flask, p-hydroxybenzaldehyde (2.34 g, 19.2 mmol), anhydrous potassium carbonate (2.91 g, 21.1 mmol) and DMF (30 ml) were added. The mixture was stirred at room temperature for 15 min, then compound **3** (4.00 g, 19.2 mmol) and potassium iodide (0.32 g, 1.9 mmol) were added and stirred for 2 h. The solution was added saturate Na_2_CO_3_ (40 ml) and EtOAc (40 ml). The organic layer was separated and washed with water (30 ml × 3), dried over anhydrous sodium sulphate, filtered, and concentrated *in vacuo* to afford crude product. The pure compound **4** was obtained by recrystallization of crude product in isopropanol.

Light yellow solid. Yield 86%; m.p. 170–172 °C; ^1^H NMR (DMSO, 400 MHz), *δ* 9.90 (s, 1H), 8.16 (dd, *J* = 8.0, 1.0 Hz, 1H), 7.90 (d, *J* = 8.8 Hz, 2H), 7.82 (ddd, *J* = 8.6, 7.2, 1.5 Hz, 1H), 7.63 (d, *J* = 7.7 Hz, 1H), 7.60–7.52 (m, 1H), 7.32 (d, *J* = 8.7 Hz, 2H), 5.45 (s, 2H), 3.62 (s, 3H)；HRMS(calcd.), *m/z*[M + H]^+^, 295.1070 (295.1077).

#### 2.1.4. Synthesis of compounds 5a–p

To a 50 ml round-bottom flask, compound **4** (1.0 g, 3.4 mmol), toluene (15 ml), corresponding aniline (6.8 mmol) and glacial acetic acid (0.02 g, 0.34 mmol) were added. The mixture was refluxed at 120 °C and water segregating reaction for 12 h. The reaction mixture was cooled to room temperature, and the solvent was evaporated. The residue was purified with methanol to afford crude product. The pure compound was obtained by recrystallization of crude product in acetonitrile.

##### 2.1.4.1. (E)-3-methyl-2-((4-((phenylimino)methyl)phenoxy)methyl)quinazolin-4(3H)-one (5a)

White solid; yield 85%; m.p. 150–152 °C; ^1^H NMR (CDCl_3_, 400 MHz), *δ* 8.38 (s, 1H), 8.30 (dd, *J* = 8.0, 0.9 Hz, 1H), 7.87 (d, *J* = 8.7 Hz, 2H), 7.75 (ddd, *J* = 18.1, 12.5, 4.4 Hz, 2H), 7.55–7.47 (m, 1H), 7.38 (t, *J* = 7.8 Hz, 2H), 7.24–7.11 (m, 5H), 5.24 (s, 2H), 3.74 (s, 3H); ^13 ^C NMR (CDCl_3_, 100 MHz), *δ* 162.3, 160.1, 159.3, 152,1, 151.2, 146.7, 134.4, 130.7, 130.5, 129.2, 127.7, 126.9, 125.8, 121.0, 120.9, 115.1, 70.1, 30.9; HRMS (calcd.), *m/z*[M + H]^+^, 370.1540(370.1550).

##### 2.1.4.2. (E)-3-methyl-2-((4-((o-tolylimino)methyl)phenoxy)methyl)quinazolin-4(3H)-one (5 b)

White solid; yield 73%; m.p. 162–164 °C; ^1^H NMR (CDCl_3_, 400 MHz), *δ* 8.33–8.26 (m, 2H), 7.88 (d, *J* = 8.7 Hz, 2H), 7.75 (ddd, *J* = 21.8, 11.5, 4.2 Hz, 2H), 7.56–7.48 (m, 1H), 7.15 (ddt, *J* = 21.5, 13.8,7.3 Hz, 5H), 6.90 (d, *J* = 7.7 Hz, 1H), 5.24 (s, 2H), 3.75 (s, 3H), 2.34 (s, 3H); ^13 ^C NMR (CDCl_3_, 100 MHz), *δ* 162.3, 160.0, 158.3, 151.2, 151.1, 146.7, 134.4, 131.9, 130.8, 130.5, 130.3, 127.7, 127.5, 126.9, 126.7, 125.5, 121.0, 117.7, 115.1, 70.1, 30.9, 17.9; HRMS (calcd.), *m/z*[M + H]^+^, 384.1698(384.1707).

##### 2.1.4.3. (E)-3-methyl-2-((4-((p-tolylimino)methyl)phenoxy)methyl)quinazolin-4(3H)-one (5c)

White solid; yield 84%; m.p. 182–184 °C; ^1^H NMR (CDCl_3_, 400 MHz), *δ* 8.39 (s, 1H), 8.29 (d, *J* = 7.9 Hz, 1H), 7.85 (d, *J* = 8.7 Hz, 2H), 7.79–7.67 (m, 2H), 7.50 (dd, *J* = 8.6, 4.0 Hz, 1H), 7.14 (dt, *J* = 12.4, 8.2 Hz, 6H), 5.23 (s, 2H), 3.74 (s, 3H), 2.35 (s, 3H); ^13 ^C NMR (CDCl_3_, 100 MHz), *δ* 162.3, 160.0, 158.4, 151.2, 149.5, 146.7, 135.6, 134.3, 130.7, 130.5, 129.8, 127.7, 127.5, 126.9, 121.0, 120.8, 115.1, 70.1, 30.8, 21.0; HRMS (calcd.), *m/z*[M + H]^+^, 384.1697(384.1707).

##### 2.1.4.4. (E)-2-((4-(((2-methoxyphenyl)imino)methyl)phenoxy)methyl)-3-methylquinazolin-4(3H)-one (5d)

White solid; yield 81%; m.p. 141–143 °C; ^1^H NMR (CDCl_3_, 400 MHz), *δ* 8.39 (s, 1H), 8.30 (dd, *J* = 8.0, 0.7 Hz, 1H), 7.89 (d, *J* = 8.7 Hz, 2H), 7.79–7.67 (m, 2H), 7.56–7.47 (m, 1H), 7.17 (ddd, *J* = 18.8, 10.0, 5.6 Hz, 3H), 7.01–6.91 (m, 3H), 5.24 (s, 2H), 3.87 (s, 3H), 3.74 (s, 3H); ^13 ^C NMR (CDCl_3_, 100 MHz), *δ* 162.3, 160.2, 152.3, 151.2, 146.7, 141.9, 134.4, 130.8, 130.7, 127.7, 127.5, 126.9, 126.5, 121.1, 121.0, 120.2, 115.0, 111.5, 70.0, 55.9, 30.9; HRMS (calcd.), *m/z*[M + H]^+^, 400.1645(400.1656).

##### 2.1.4.5. (E)-2-((4-(((4-methoxyphenyl)imino)methyl)phenoxy)methyl)-3-methylquinazolin-4(3H)-one (5e)

White solid; yield 88%; m.p. 180–182 °C; ^1^H NMR (CDCl_3_, 400 MHz), *δ* 8.40 (s, 1H), 8.29 (dd, *J* = 8.0, 1.0 Hz, 1H), 7.85 (d, *J* = 8.8 Hz, 2H), 7.80–7.65 (m, 2H), 7.55–7.46 (m, 1H), 7.25–7.16 (m, 2H), 7.13 (d, *J* = 8.8 Hz, 2H), 6.97–6.85 (m, 2H), 5.23 (s, 2H), 3.81 (s, 3H), 3.74 (s, 3H); ^13 ^C NMR (CDCl_3_, 100 MHz), *δ* 162.3, 159.82, 158.1, 157.3, 151.2, 146.7, 145.0, 134.4, 130.8, 130.4, 127.7, 127.5, 126.9, 122.1, 121.0, 115.1, 114.4, 70.1, 55.5, 30.9; HRMS (calcd.), *m/z*[M + H]^+^, 400.1645(400.1656).

##### 2.1.4.6. (E)-2-((4-(((2-fluorophenyl)imino)methyl)phenoxy)methyl)-3-methylquinazolin-4 (3H)-one (5f)

White solid; yield 76%; m.p. 143–145 °C; ^1^H NMR (CDCl_3_, 400 MHz), *δ* 8.44 (s, 1H), 8.30 (dd, *J* = 8.0, 0.8 Hz, 1H), 7.89 (d, *J* = 8.8 Hz, 2H), 7.80–7.69 (m, 2H), 7.56–7.48 (m, 1H), 7.18–7.09 (m, 6H), 5.25 (s, 2H), 3.75 (s, 3H); ^13 ^C NMR (CDCl_3_, 100 MHz), *δ* 162.3, 161.8, 160.4, 156,5, 154.0, 151.1, 146.7, 140.0, 134.4, 130.9, 130.3, 127.7, 127.5, 126.9, 124.5, 122.0, 121.0, 116.3, 116.1, 115.1, 70.1, 30.9; HRMS (calcd.), *m/z*[M + H]^+^, 388.1445(388.1456).

##### 2.1.4.7. (E)-2-((4-(((3-fluorophenyl)imino)methyl)phenoxy)methyl)-3-methylquinazolin-4(3H)-one (5 g)

White solid; yield 72%; m.p. 168–170 °C; ^1^H NMR (CDCl_3_, 400 MHz), *δ* 8.35 (s, 1H), 8.30 (d, *J* = 7.9 Hz, 1H), 7.86 (d, *J* = 8.7 Hz, 2H), 7.75 (ddd, *J* = 19.2, 13.1, 4.7 Hz, 2H),7.51 (dd, *J* = 10.9, 4.1 Hz, 1H), 7.35–7.28 (m, 1H), 7.16 (d, *J* = 8.7 Hz, 2H), 6.93 (ddd, *J* = 18.7, 12.0,4.7 Hz, 3H), 5.25 (s, 2H), 3.75 (s, 3H); ^13 ^C NMR (CDCl_3_, 100 MHz), *δ* 164.5, 162.3, 160.3, 160.1, 153.9, 151.1, 146.7, 134.4, 130.9, 130.1, 127.7, 127.5, 126.9, 121.0, 116.8, 115.2, 112.5, 112.3, 108.2, 107.9, 70.1, 30.9; HRMS (calcd.), *m/z*[M + H]^+^, 388.1446(388.1456).

##### 2.1.4.8. (E)-2-((4-(((4-fluorophenyl)imino)methyl)phenoxy)methyl)-3-methylquinazolin-4(3H)-one (5 h)

White solid; yield 75%; m.p. 200–202 °C; ^1^H NMR (CDCl_3_, 400 MHz), *δ* 8.36 (s, 1H), 8.30 (d, *J* = 8.0 Hz, 1H), 7.86 (d, *J* = 8.5 Hz, 2H), 7.75 (dt, *J* = 19.0, 7.6 Hz, 2H),7.52 (t, *J* = 7.5 Hz, 1H), 7.20–7.11 (m, 4H), 7.06 (t, *J* = 8.5 Hz, 2H), 5.25 (s, 2H), 3.75 (s, 3H); ^13 ^C NMR (CDCl_3_, 100 MHz), *δ* 162.3, 160.1, 159.9, 159.1, 151.2, 148.1, 146.7, 134.4, 130.6, 130.4, 127.7, 127.5, 126.9, 122.3, 122.2, 121.0, 116.0, 115.8, 115.1, 70.1, 30.9; HRMS (calcd.), *m/z*[M + H]^+^, 388.1448(388.1456).

##### 2.1.4.9. (E)-2-((4-(((3-chlorophenyl)imino)methyl)phenoxy)methyl)-3-methylquinazolin-4(3H)-one (5i)

White solid; yield 80%; m.p. 179–181 °C; ^1^H NMR (CDCl_3_, 400 MHz), *δ* 8.34 (s, 1H), 8.33–8.26 (m, 1H), 7.86 (d, *J* = 8.7 Hz, 2H), 7.79–7.69 (m, 2H), 7.55–7.48 (m, 1H), 7.29 (t, *J* = 7.9 Hz, 1H), 7.17 (t, *J* = 8.4 Hz, 4H), 7.06 (d, *J* = 8.2 Hz, 1H), 5.25 (s, 2H), 3.75 (s, 3H); ^13 ^C NMR (CDCl_3_, 100 MHz), *δ* 162.3, 160.4, 160.2, 153.4, 151.1, 146.7, 134.7, 134.4, 130.9, 130.2, 127.7, 127.5, 126.9, 125.7, 121.0, 120.9, 119.5, 115.2, 70.1, 30.9; HRMS (calcd.), *m/z*[M + H]^+^, 404.1150(404.1160).

##### 2.1.4.10. (E)-2-((4-(((3-bromophenyl)imino)methyl)phenoxy)methyl)-3-methylquinazolin-4(3H)-one (5j)

White solid; yield 85%; m.p. 176–178 °C; ^1^H NMR (CDCl_3_, 400 MHz), *δ* 8.34 (s, 1H), 8.30 (d, *J* = 7.9 Hz, 1H), 7.86 (d, *J* = 8.6 Hz, 2H), 7.80–7.68 (m, 2H), 7.52 (t, *J* = 7.2 Hz, 1H), 7.33 (d, *J* = 6.6 Hz, 2H), 7.24 (t, *J* = 8.1 Hz, 1H), 7.14 (dd, *J* = 19.3, 8.2 Hz, 3H), 5.26 (s, 2H), 3.75 (s, 3H); ^13 ^C NMR (CDCl_3_, 100 MHz), *δ* 162.3, 160.4, 160.2, 153.6, 151.1, 146.7, 134.4, 130.9, 130.4, 130.1, 128.6, 127.7, 127.5, 126.9, 123.7, 122.8, 121.0, 120.0, 115.2, 70.1, 30.9; HRMS (calcd.), *m/z*[M + H]^+^, 448.0647(448.0655).

##### 2.1.4.11. (E)-2-((4-(((3,4-difluorophenyl)imino)methyl)phenoxy)methyl)-3-methylquinazolin-4(3H)-one (5k)

White solid; yield 80%; m.p. 152–154 °C; ^1^H NMR (CDCl_3_, 400 MHz), *δ* 8.34 (s, 1H), 8.32–8.26 (m, 1H), 7.86 (d, *J* = 8.8 Hz, 2H), 7.76 (ddd, *J* = 20.4, 13.6, 4.4 Hz, 2H), 7.56–7.49 (m, 1H), 7.20–7.10 (m, 3H), 7.03 (ddd, *J* = 11.4, 7.2, 2.5 Hz, 1H), 6.96–6.89 (m, 1H), 5.26 (s, 2H), 3.76 (s, 3H); ^13 ^C NMR (CDCl_3_, 100 MHz), *δ* 162.3, 160.4, 159.9, 151.1, 146.7, 134.4, 130.8, 130.0, 127.7, 127.5, 126.9, 121.0, 117.5, 117.3, 117.0, 116.9, 116.8, 115.2, 110.0, 109.8, 70.1, 30.9; HRMS (calcd.), *m/z*[M + H]^+^, 406.1354(406.1362).

##### 2.1.4.12. (E)-2-((4-(((3,5-difluorophenyl)imino)methyl)phenoxy)methyl)-3-methylquinazolin-4(3H)-one (5 l)

White solid; yield 87%; m.p. 220–221 °C; ^1^H NMR (DMSO-*d_6_*, 400 MHz), *δ* 8.58 (s, 1H), 8.17 (dd, *J* = 8.0, 1.1 Hz, 1H), 7.91 (d, *J* = 8.9 Hz, 2H), 7.83 (dd, *J* = 11.1, 4.2 Hz, 1H), 7.65 (d, *J* = 7.7 Hz, 1H),7.60–7.53 (m, 1H), 7.27 (d, *J* = 8.8 Hz, 2H), 7.09 (td, *J* = 9.4, 4.7 Hz, 1H), 7.03–6.96 (m, 2H), 5.42 (s, 2H), 3.62 (s, 3H); ^13 ^C NMR (DMSO-*d_6_*, 100 MHz), *δ* 162.0, 161.7, 160.8, 160.3, 159.6, 152.7, 148.5, 146.9, 134.9, 130.9, 130.1, 127.8, 127.6, 126.7, 123.2, 123.1, 120.8, 116.4, 116.2, 115.8, 69.0, 30.4; HRMS (calcd.), *m/z*[M + H]^+^, 406.1355(406.1362).

##### 2.1.4.13. (E)-2-((4-(((2,6-difluorophenyl)imino)methyl)phenoxy)methyl)-3-methylquinazolin-4(3H)-one (5 m)

White solid; yield 80%; m.p. 181–183 °C; ^1^H NMR (CDCl_3_, 400 MHz), *δ* 8.53 (s, 1H), 8.30 (d, *J* = 7.8 Hz, 1H), 7.91 (d, *J* = 8.5 Hz, 2H), 7.81–7.66 (m, 2H), 7.52 (t, *J* = 7.3 Hz, 1H), 7.16 (d, *J* = 8.5 Hz, 2H), 7.09–6.99 (m, 1H), 6.94 (t, *J* = 7.7 Hz, 2H), 5.26 (s, 2H), 3.75 (s, 3H); ^13 ^C NMR (CDCl_3_, 100 MHz), *δ* 165.7, 162.3, 160.7, 156.5, 156.4, 154.0, 153.9, 151.1, 146.7, 134.4, 131.0, 130.1, 127.7, 127.5, 126.9, 124.9, 121.0, 115.1, 111.9, 111.7, 70.1, 30.9; HRMS (calcd.), *m/z*[M + H]^+^, 406.1351(406.1362).

##### 2.1.4.14. (E)-2-((4-(((2,4-difluorophenyl)imino)methyl)phenoxy)methyl)-3-methylquinazolin-4(3H)-one (5n)

White solid; yield 72%; m.p. 181–183 °C; ^1^H NMR (CDCl_3_, 400 MHz), *δ* 8.43 (s, 1H), 8.34–8.26 (m, 1H), 7.88 (dd, *J* = 5.3, 3.4 Hz, 2H), 7.82–7.63 (m, 2H), 7.59–7.45 (m, 1H), 7.15 (dd, *J* = 5.3, 3.4 Hz, 3H), 6.96–6.76 (m, 2H), 5.25 (s, 2H), 3.75 (s, 3H); ^13 ^C NMR (CDCl_3_, 100 MHz), *δ* 162.3, 161.6, 160.4, 154.0, 151.1, 146.7, 134.4, 130.9, 130.2, 127.7, 127.5, 126.9, 122.4, 121.0, 115.2, 111.5, 111.2, 104.9, 104.6, 104.4, 70.1, 30.9; HRMS (calcd.), *m/z*[M + H]^+^, 406.1355(406.1362).

##### 2.1.4.15. (E)-2-((4-(((3-chloro-4-fluorophenyl)imino)methyl)phenoxy)methyl)-3-methyl quinazolin-4(3H)-one (5o)

White solid; yield 80%; m.p. 188–189 °C; ^1^H NMR (CDCl_3_, 400 MHz), *δ* 8.34 (s, 1H), 8.30 (d, *J* = 7.9 Hz, 1H), 7.85 (d, *J* = 8.6 Hz, 2H), 7.78 (t, *J* = 7.5 Hz, 1H), 7.72 (d, *J* = 8.0 Hz, 1H), 7.53 (t, *J* = 7.4 Hz, 1H), 7.27–7.21 (m, 1H), 7.20–7.04 (m, 4H), 5.26 (s, 2H), 3.76 (s, 3H); ^13 ^C NMR (CDCl_3_, 100 MHz), *δ* 162.3, 160.4, 160.0, 151.1, 148.7, 146.7, 134.4, 130.8, 130.0, 127.7, 127.5, 126.9, 122.6, 121.2, 121.0, 120.9, 120.8, 117.0, 116.7, 115.2, 70.1, 30.9; HRMS (calcd.), *m/z*[M + H]^+^, 422.1054(422.1066).

##### 2.1.4.16. (E)-2-((4-(((4-chloro-3-(trifluoromethyl)phenyl)imino)methyl)phenoxy)methyl)-3-methylquinazolin-4(3H)-one (5p)

White solid; yield 81%; m.p. 163–164 °C; ^1^H NMR (CDCl_3_, 400 MHz), *δ* 8.36 (s, 1H), 8.29 (dd, *J* = 8.0,0.8 Hz, 1H), 7.87 (d, *J* = 8.7 Hz, 2H), 7.79–7.68 (m, 2H), 7.51 (dt, *J* = 11.4, 5.0 Hz, 3H), 7.29–7.23 (m, 1H), 7.17 (d, *J* = 8.7 Hz, 2H), 5.26 (s, 2H), 3.75 (s, 3H); ^13 ^C NMR (CDCl_3_, 100 MHz), *δ* 162.3, 160.8, 160.6, 151.0, 150.7, 146.7, 134.4, 132.2, 131.0, 129.8, 129.1, 128.8, 128.7, 127.7, 127.5, 126.9, 125.0, 124.1, 121.0, 120.4, 120.3, 120.3, 120.2, 115.3, 70.1, 30.9; HRMS (calcd.), *m/z*[M + H]^+^, 472.1022(472.1034).

### 2.2. *In vitro* EGFR^wt^-TK assay

Recombinant EGFR was purchased from Sino Biology Inc. Antiphosphotyrosine mouse mAb was purchased from PTM Bio. The effects of compounds on the activity of wild type EGFR tyrosine kinase were determined by enzyme-linked immunosorbent assays (ELISAs) with recombinant EGFR according to reported methods.

### 2.3 *In vitro* activity assay at cell level

#### 2.3.1. Cell culture

A549 (Human non-small cell lung cancer cell line) cell line was purchased from the Shanghai Cell Bank of the Chinese Academy of Sciences; PC-3 (Human prostate cancer cell line) cell line was donated by the Key Laboratory of Natural Product Chemistry of the Chinese Academy of Sciences of Guizhou Province; SMMC-7721 (Human liver cancer cell line) cell line was donated by the Department of Immunology, the Third Military Medical University; NRK 52E (Normal rat kidney cell line) cell line was donated by Guizhou Medical University; All cell lines were kept by the laboratory. Cells were maintained in RPMI 1640 or DMEM complete medium.

#### 2.3.2. Cytotoxicity evaluation (MTT assay)

*In vitro* cytotoxicity of synthesised compounds against three kinds of human tumour cell lines (PC-3, SMMC-7721, A549) along with normal rat kidney cells (NRK 52E) was determined by MTT assay as described in previous articles. 5-Fluorouracil and Gefitinib were used as positive controls.

#### 2.3.3. Cell apoptosis analysis

The apoptosis of tumour cells (A549) treated by different concentrations of compound **5k**, was measured with Annexin V–FITC/PI apoptosis detection kit (Solarbio, Beijing, China), according to instructions of kit, and detected by BD Accuri C6 flow cytometry (American BD Corporation Shanghai Co., Ltd.)

#### 2.3.4. Cell cycle analysis

The distribution of cell cycle for A549 treated by different concentrations of compound **5o**, was measured with Annexin V–FITC/PI cell cycle detection kit (Solarbio, Beijing, China), according to instructions of kit, and detected by BD Accuri C6 flow cytometry (American BD Corporation Shanghai Co., Ltd.)

### 2.4. Molecular docking

The possible binding modes of all designed compound with EGFR were predicted by molecular docking with Autodock Vina. Base on the trajectories of 10 ns MD simulation for each candidate conformation from docking, MM/GBSA method was used to estimate the binding affinity between EGFR and ligand for identifying the most possible binding modes. X-ray crystal structures of EGFR in both “active” (PDB entry 1M17) and “inactive” (PDB entry 4HJO) states were used for identifying candidate binding modes.

### 2.5. ADMET studies

All the compounds were built and full minimised using CHARMM force field using Discovery Studio 2.5 software (Accelrys, Inc., San Diego, Calif.). The low-energy conformers of all the compounds were used to calculate the ADMET properties. The parameters of ADMET were judged according to the levels shown for each parameter. ADMET aqueous solubility levels were six as 0 for extremely low, 1 for no; very low, but possible; 2 for yes, low; 3 for yes, good; 4 for yes, optimal, 5 for no, too soluble and 6 for molecules with one or more unknown AlogP98 types. Intestinal absorption levels (HIA) were 0, 1, 2, and 3 which showed good, moderate, low, or very low absorption, respectively. Cytochrome P450 (CYP2D6) inhibition, hepatotoxicity were shown as 1 for the inhibitor and toxic or 0 for non-inhibitor and non-toxic, respectively. Plasma protein binding (PPB) was 0, 1, 2 which showed binding is < 90%, > 90%, or > 95%. Blood brain barrier permeability (BBB) were classified by 0 for very high, 1 for high, 2 for medium, 3 for low and 4 for undefined.

## 3. Results and discussions

### 3.1 Chemistry

The target compounds (**5a–5p**) were synthesised from the starting material isatoic anhydride in a sequence of reactions and characterised by NMR and HRMS. As shown in Scheme 1, compound **2** and **3** were obtained according to the reported method^19^. Compound **3** reacted with 4-hydroxy-benzaldehyde under the K_2_CO_3_/KI/DMF conditions to give the compound **4** with 86% yield. And then, compound **4** reacted with corresponding phenylamine to get the final targets. The compounds **5a–p** were purified by recrystallization in acetonitrile.

### 3.2. *In vitro* EGFR kinase inhibitory activity and antitumor activity of target quinazolinone derivatives

In order to test our rationality of the design, we took **5a** which bears a hydrogen moiety at R as our initial compound to evaluate the inhibitory activity against EGFR^wt^-TK. The result revealed that **5a** have poor activity with an inhibition rate of 1.91% at 1 μM (Table 1, **5a**). Then the group of R was replaced with methyl, methoxy and halogen, respectively (Table 1, 5b**–**j). Fortunately, the compound **5g** reached in 64.95% at 1 μM against the kinase. As a comparison, in order to investigate the influence of flouro-group on the inhibitory activity, we used difluoro-group (Table 1, 5k-o), and trifluoromethyl group (Table 1, **5p**) to replace fluoro moiety. As shown in Table 1, the best compounds **5k** and **5l** were tested with 63.37% and 61.89% by ELISA kinase assay^20^ at the concentration of 1 μM. 5-Fluorouracil and Gefitinib were used as positive control with 63.42% and 67.85% at 1 μM, respectively.

**Table 1.** *In vitro* inhibitory activities of target compounds for EGFR^wt^-TK and human cancer cell lines A549, PC-3 and SMMC-7721^a^.
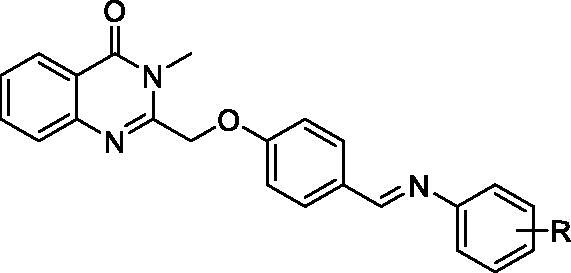

Comp.	R	EGFR^wt^-TK inhibition rate (%, 1 μM)	Inhibition rate (%, 10 μM)		
			A549	PC-3	SMMC-7721
**5a**	H	1.91 ± 0.91	15.38 ± 1.52	7.36 ± 2.38	15.06 ± 5.91
**5b**	2-CH_3_	2.57 ± 1.25	14.49 ± 4.09	23.94 ± 8.59	24.34 ± 1.70
**5c**	4-CH_3_	50.75 ± 3.29	12.09 ± 2.00	7.46 ± 0.52	31.77 ± 9.91
**5d**	2-OCH_3_	38.73 ± 1.83	20.84 ± 4.81	38.24 ± 1.40	2.78 ± 4.23
**5e**	4-OCH_3_	53.65 ± 1.25	25.95 ± 7.46	40.20 ± 3.57	20.09 ± 9.11
**5f**	2-F	1.31 ± 3.64	16.31 ± 1.43	5.46 ± 0.28	2.65 ± 1.81
**5g**	3-F	64.95 ± 1.51	15.59 ± 2.56	4.76 ± 0.51	35.65 ± 1.27
**5h**	4-F	56.21 ± 2.16	14.79 ± 5.06	14.81 ± 2.30	22.86 ± 1.66
**5i**	3-Cl	1.04 ± 0.91	23.26 ± 7.52	9.60 ± 2.69	13.14 ± 2.92
**5j**	3-Br	53.26 ± 1.58	18.02 ± 6.69	17.92 ± 6.12	18.62 ± 1.51
**5k**	3,4-di-F	63.37 ± 1.83	27.01 ± 1.63	57.47 ± 2.77	34.93 ± 4.72
**5l**	3,5-di-F	61.89 ± 1.51	31.21 ± 2.49	54.28 ± 7.59	41.00 ± 2.63
**5m**	2,6-di-F	25.84 ± 1.92	15.16 ± 3.61	26.80 ± 0.59	15.12 ± 5.79
**5n**	2,4-di-F	48.40 ± 0.35	33.29 ± 3.63	25.84 ± 4.84	11.51 ± 2.87
**5o**	3-Cl-4-F	16.72 ± 2.99	18.72 ± 9.78	13.91 ± 0.92	24.46 ± 1.37
**5p**	4-Cl-3-CF_3_	4.32 ± 1.92	15.60 ± 2.66	22.55 ± 1.28	17.50 ± 2.67
Gefitinib		67.85 ± 0.85	27.41 ± 4.38	30.66 ± 7.58	21.71 ± 1.68
5-Fluorouracil		63.42 ± 1.69	40.37 ± 1.15	29.32 ± 1.24	29.08 ± 2.75

^a^ The values are mean ± SD of three replicates.

To test the anticancer activity of synthesised compounds, we evaluated the anti-proliferative activity of compounds **5a–p** against three EGFR over-expressed human tumour cell lines derived from human non-small cell lung cancer (A549), prostate cancer (PC-3), and liver cancer (SMMC-7721) by MTT assay. 5-Fluorouracil and Gefitinib were also used as positive controls. As shown in Table 1, part of the novel derivatives exhibited potent anti-cancer activities against selected cancer cell lines with 10 μM. Against A549 cells, compounds **5l** and **5n** (with inhibitions of 31.21% and 33.29%, respectively) were more potent than Gefitinib (27.41%). The inhibitory efficacy of compounds **5k** and **5l** against SMMC-7721 and PC-3 cells was higher than Gefitinib and 5-Fluorouracil.

Based on the initial result of EGFR^wt^-TK and three tumour cell assays, we obtain the preliminary structure-activity relationship (SAR) of quinazolinone derivatives. It is indicated that this type of substitution in phenyl ring is essential for anticancer activity. For anti-tumour activity, the effective substitution order should be F > OCH_3_ ≈ Br > CH_3_ > H ≈ Cl. Also, the more effective substitutions of the phenyl ring are either 3-F, 3,4-di-F or 3,5-di-F group(s). Thus for good anti-cancer activity, the 3-position of phenyl ring should carry fluoro group.

To further evaluating bioactivities, the IC_50_ values of compounds **5g**, **5k** and **5l** to EGFR^wt^-TK and three human cancer cell lines (A549, PC-3 and SMMC-7721) were evaluated, using 5-fluorouracil and Gefitinib as positive controls. As shown in Table 2, compound **5k** inhibited EGFR activity with an IC_50_ value of 0.010 ± 0.001 μM and was similar to Gefitinib. However, compounds **5g** and **5l** did not cause significant EGFR inhibition with 0.047 ± 0.004 μM and 0.54 ± 0.031 μM. On the other hand, compound **5k** inhibited anti-cancer cells (A549, PC-3 and SMMC-7721) activity with IC_50_ values of 12.30 ± 4.12 μM, 17.08 ± 3.61 μM and 15.68 ± 1.64 μM. These data indicate that **5p** was worth to further investigation.

**Table 2.** IC_50_ values for EGFR^wt^-TK and human cancer cell lines A549, PC-3 and SMMC-7721^a^.

Comp.	R	IC_50_ (μM)			
		EGFR^wt^-TK	A549	PC-3	SMMC-7721
**5g**	3-F	0.047 ± 0.004	19.73 ± 2.34	30.06 ± 1.86	35.92 ± 5.85
**5k**	3,4-di-F	0.010 ± 0.001	12.30 ± 4.12	17.08 ± 3.61	15.68 ± 1.64
**5l**	3,5-di-F	0.54 ± 0.031	7.22 ± 3.51	13.29 ± 1.12	6.04 ± 0.55
Gefitinib		0.0061 ± 0.0003	5.48 ± 1.06	30.40 ± 0.34	9.85 ± 3.44
5-Fluorouracil		0.14 ± 0.011	1.53 ± 0.72	22.46 ± 0.74	11.37 ± 1.95

^a^ The values are mean ± SD of three replicates.

### 3.3. *In vitro* cytotoxicity of target quinazolinone derivatives on normal cells

**Figure 3.**
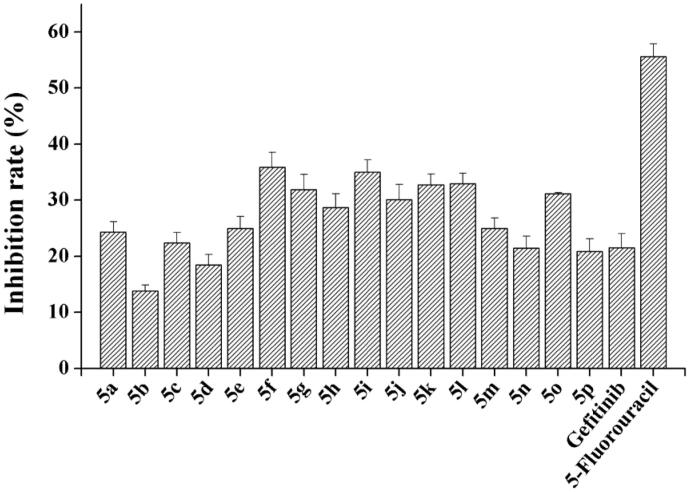
*In vitro* cytotoxicity of target compounds on normal rat kidney cell NRK-52E.

As the preferential selectivity for cancer cell is critical for antitumor drugs, the cytotoxicity of these novel compounds to normal rat kidney cell line (NRK-52E) was tested by MTT assay. All target compounds have significantly lower cytotoxicity than 5-fluorouracil (Figure 3). The cytotoxicity of most compounds was similar to Gefitinib, even the partial compounds were much lower.

### 3.4. Effects of compound 5k on cell apoptosis of A549 cell line

**Figure 4.**
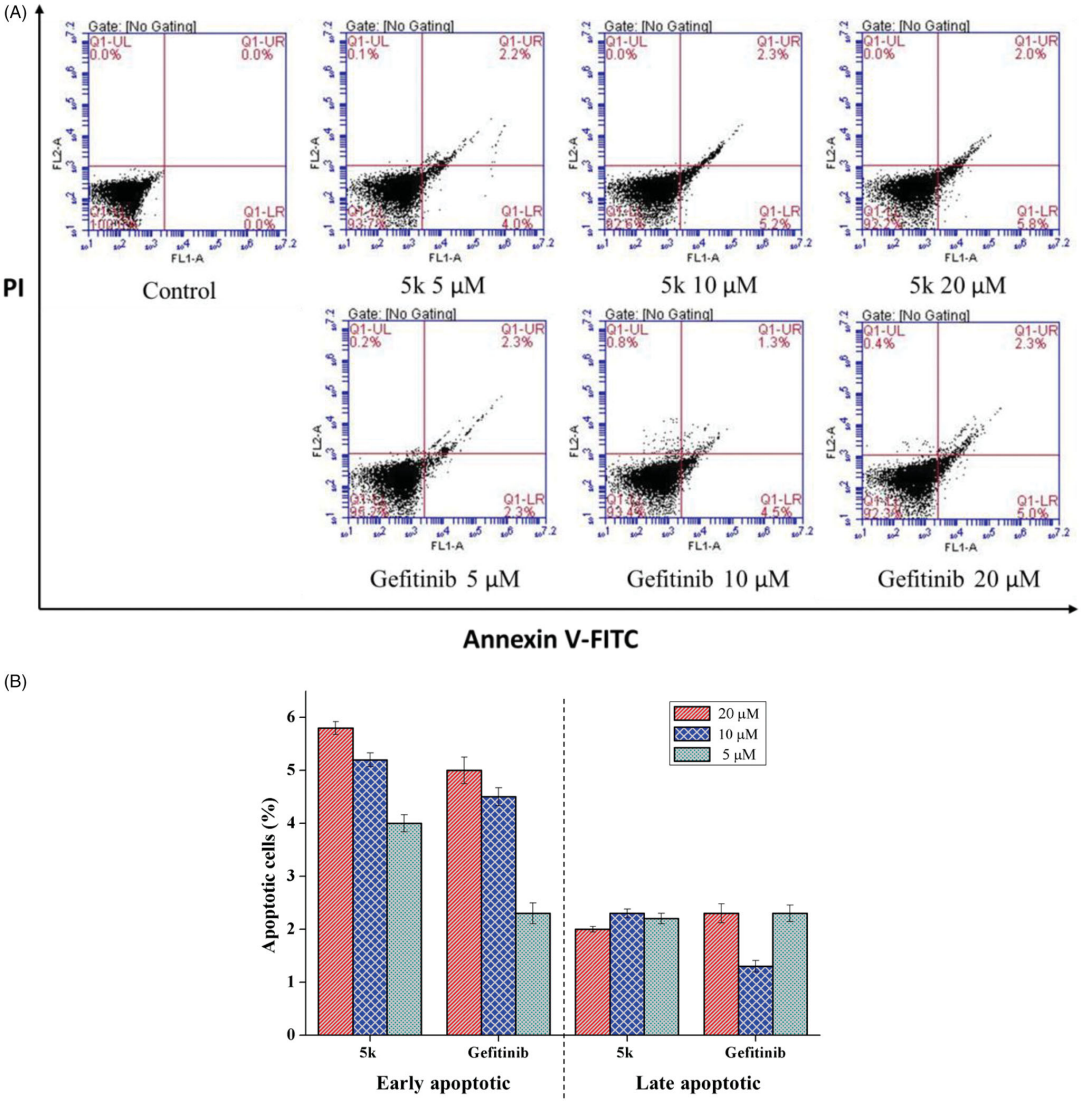
Compound **5k** induced cell apoptosis in Annexin V-FITC assay. (A) Density plot were obtained by flow cytometry, Gefitinib was used as reference drug. (B) Total apoptotic cells (%) at various concentrate of **5k** and Gefitinib.

Apoptosis is a physiological process that functions as an essential mechanism of tissue homeostasis and is regarded as the preferred way to eliminate unwanted cells. Most of the available anti-cancer drugs mediate their effects *via* induction of apoptosis in cancer cells^21^. To further investigate the effect of **5k** on tumour cell, apoptosis of A549 cell treated with different concentrations of **5k** for 48 h was analysed by flow cytometry. Herein, Gefitinib was used as positive control. After treatment with **5k** or Gefitinib, the number of apoptotic cells increased significantly (Figure 4). The trend of early apoptosis rate showed that **5k** induces cellular apoptosis in a concentration-dependent way. The results also showed compound **5k** has better ability to induce early apoptosis than gefitinib at 5 μM, 10 μM and 20 μM (Figure 4(B)). The difference in the total numbers of apoptotic cells between samples treated with **5k** or Gefitinib at the same drug concentration decreased with increasing drug concentration, indicating that **5o** has a better ability to induce apoptosis at low concentrations than Gefitinib.

### 3.5. Effects of compound 5k on cell cycle of A549 cell line

**Figure 5.**
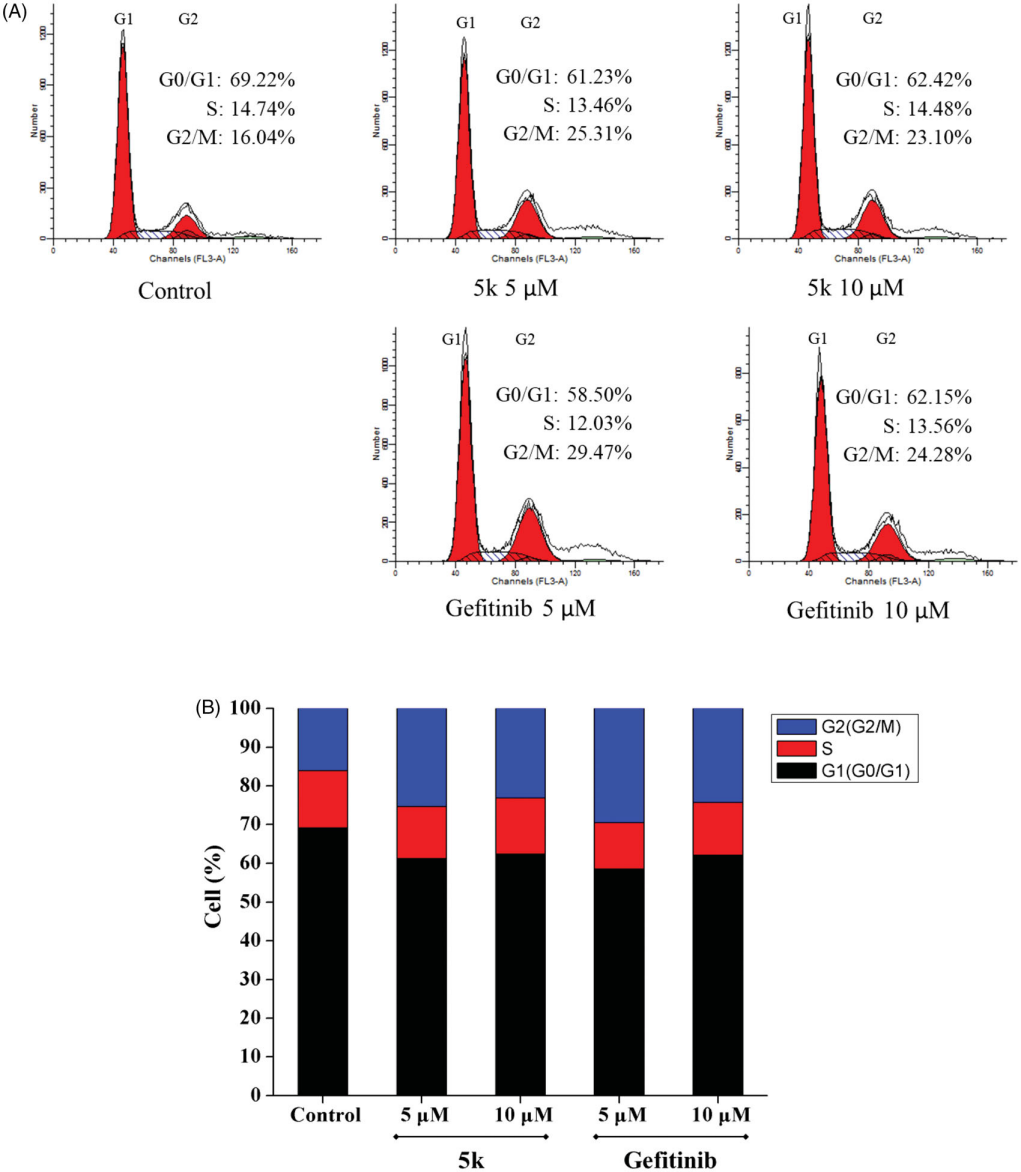
Effect of compound **5k** on the cell cycle phase distribution in A549 cells (A) Profiles were obtained by FACS. The percentages for different phases of cell cycle were illustrated in the histogram. (B) A549 cells were cultured in the presence of different concentrations of **5k** (5 μM and 10 μM) or Gefitinib (5 μM and 10 μM) for 48 h, harvested, fixed, and labelled with PI, then analysed by FACS. Percentage of cells in G0/G1, S and G2/M phases are indicated.

Most cytotoxic agents cause cell cycle arrest, either as the direct consequence of regulating cell cycle regulators, or as the secondary effect of modifying other cell components^22^. In order to study the effect of compound **5k** on cell cycle, A549 cells were treated by compound **5k** or Gefitinib for 48 h, then the cells were strained by PI and examined using flow cytometry. Comparing with control A549 cells that consist of 16.04% of G2 phase cells, the G2 phase percentage of A549 cells treated with compound **5k** and Gefitinib at 5 μM and 10 μM increased to 25.31% and 29.47% and 23.10%, 24.28%, respectively (Figure 5) accompanied by a decrease in the S and G0/G1 cells. The results indicated A549 cells could be arrested in the G2/M phase by compound **5k**. However, at 10 μM of compound exposure, G2/M arrest decreased, A549 cells would undergo significant apoptosis. These data suggest that the G2/M phase arrest in A549 cells by compound **5k** and Gefitinib might be in parallel to the induction of apoptosis.

### 3.6. Molecular docking studies

To investigate the detailed protein-ligand interaction between EGFR and the novel scaffold, the possible binding modes were predicted by molecular docking. Previous structural studies showed that the “active” or “inactive” state of EGFR tyrosine kinase has different preference of ligand binding profile, so X-ray crystal structures of EGFR in both “active” (PDB entry 1M17) and “inactive” (PDB entry 4HJO) states were used for identifying candidate binding modes^23^.

**Figure 6.**
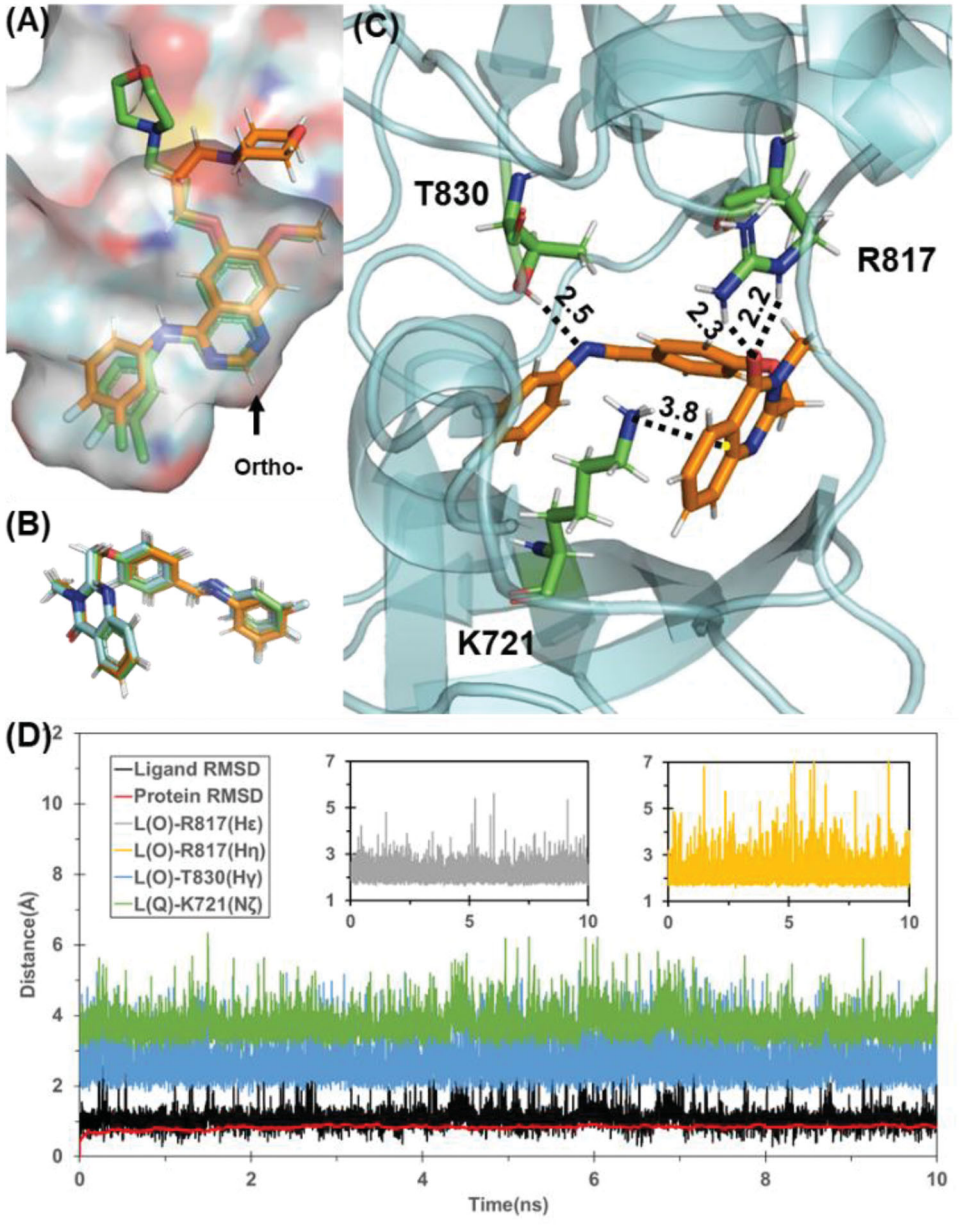
(A) Superimpose of computational predicted gefitinib binding conformation (gold) with conformation from X-ray crystal structure (green). (B) Predicted binding conformations of compound **5 g**(green), **5k**(cyan), and **5 l**(gold). (C) Binding mode of compound **5k**. Residues T830, R817, and K721 are shown in stick style and Coloured in green. Hydrogen bond and cation-π interaction are represented as dashed lines with labelled distance in unit of Å. (D) Tracked changes for the positional root-mean-square deviations (RMSD) for the EGFR (red curve) and compound **5k** (black curve) during MD simulation. L(O) and L(Q) represents the oxygen in quinazolinone and quinazolinone itself, respectively.

Based on data shown in Table S1, the predicted binding energy of all the designed compounds **5a–p** with inactive state EGFR are in the range of −40 to −45 kcal/mol, whereas the binding energy for active state EGFR falls to the range of −22 to −36 kcal/mol, which suggests that these compounds are likely to bind with EGFR in inactive state. In contrast, the predicted binding affinity for Gefitinib with EGFR in two states are very similar (−45.7 kcal/mol for active state and −45.8 kcal/mol for inactive state), which is consistent with previously reported work^24^. With the same docking and MD simulation protocol for designed scaffolds and Gefitinib, the binding mode of Gefitinib was successfully reconstructed with quinazoline scaffold well-fitted to the X-ray crystal structure of EGFR/Gefitinib complex (Figure 6(A)). Obviously, the ortho-substituted quinazolinone derivatives could not adopt similar binding mode of Gefitinib because the corresponding substitution site of Gefitinib is closely face to protein. Interestingly, all of these compounds **5a–p** bind to EGFR with a similar binding mode depicted in Figure 6(B). Take compound **5k** as example, the quinazolinone moiety formed cation-π interaction with the residue K721 (Figure 6(C,D)), which is a key residue for ATP-binding of EGFR. Besides, **5k** also formed stable hydrogen bonds with R817 and T830 during the 10 ns MD simulation. The stable protein-ligand binding during MD simulation for these compounds also results in a small standard deviation for the energy prediction (±3 kcal/mol), Although it is still not feasible to rank these compounds by the binding energy within a narrow range of −40 to −45 kcal/mol, the computational prediction indicates that the binding affinity of these designed compounds are comparable with gefitinib, which also suggests the common scaffold of these compounds may be a promising lead scaffold for designing novel EGFR-TK.

### 3.7. ADMET studies

Furthermore, Discovery Studio 2.5 software (Accelrys, Inc., San Diego, Calif.) was used to predict the physicochemical and ADMET properties of the synthesised compounds **5a–p**. As shown in Table 3, aqueous solubility logarithmic levels of the compounds **5a–o** were 2, suggesting that these compounds are the same with Gefitinib. CYP2D6 binding predicts are non-inhibitor of cytochrome P450 enzyme. The compounds **5l**, **5m** and **5p** were probably less toxic than Gefitinib, and the others were similar with Gefitinib. Furthermore, the calculated log *p* values 4.34 and 4.54 were closed to Gefitinib (4.20), suggesting that the fluoro- group or difluoro-group on phenyl were well for physicochemical properties. This result is consistent with the previous structure-activity relationship.

**Table 3.** ADMET properties of all compounds.

Comp.	R	Solubility	Absorption	CYP2D6	Hepatotoxicity	PPB	BBB	AlogP98
**5a**	H	2	0	1	1	2	1	4.13
**5b**	2-CH_3_	2	0	1	1	2	1	4.62
**5c**	4-CH_3_	2	0	1	1	2	1	4.62
**5d**	2-OCH_3_	2	0	1	1	2	1	4.11
**5e**	4-OCH_3_	2	0	1	1	2	1	4.11
**5f**	2-F	2	0	1	1	2	1	4.34
**5g**	3-F	2	0	1	1	2	1	4.34
**5h**	4-F	2	0	1	1	2	1	4.34
**5i**	3-Cl	2	0	1	1	2	1	4.79
**5j**	3-Br	2	0	1	1	2	1	4.88
**5k**	3,4-di-F	2	0	1	1	2	1	4.54
**5l**	3,5-di-F	2	0	0	1	2	1	4.54
**5m**	2,6-di-F	2	0	0	1	2	1	4.54
**5n**	2,4-di-F	2	0	1	1	2	1	4.54
**5o**	3-Cl-4-F	2	0	1	1	2	1	5.00
**5p**	4-Cl-3-CF_3_	1	0	0	1	2	0	5.74
**Gefitinib**		2	0	1	0	1	1	4.20

## 4. Conclusion

In summary, a series of novel 3-methyl-quinazolinone derivatives were designed and synthesised. *In vitro* biological activities of novel compounds on enzyme level and cell level were evaluated. The enzyme inhibition assay showed that **5g**, **5k,** and **5l** have the good inhibition for EGFR^wt^-TK. Two compounds **5k** and **5l** were identified to show superior biological activities than Gefitinib and 5-fluorouracil in both PC-3 and SMMC-7721 cell lines. Importantly, the best compound **5k** displayed 10 nM IC_50_ value against EGFR^wt^-TK activity. Apoptosis analysis in A549 cell line suggested that **5k** delayed cell cycle progression by arresting cells in the G2/M phase of the cell cycle, retarding cell growth. Furthermore, molecular docking, molecular dynamics simulation, and MM/GBSA estimation were performed to determine possible binding modes of these compounds with EGFR. This design offered an option for the optimisation and development of quinazolinone-based EGFR inhibitors. Other further studies on compounds **5k** and **5l** are going on in our lab.

## Supplementary Material

Supplemental Material
