# Supplementary material for: Design, synthesis and in vitro biological evaluation of quinazolinone derivatives as EGFR inhibitors for antitumor treatment
Source: J Enzyme Inhib Med Chem. 2020 Jan 22;35(1):555–64. doi: 10.1080/14756366.2020.1715389 (PMC7006757; doi:10.1080/14756366.2020.1715389)
Supplement: Supplemental Material [file IENZ_A_1715389_SM2676.pdf]

Supporting Information

Design, synthesis and in vitro biological evaluation of quinazolinone derivatives as EGFR inhibitors for antitumor treatment

Yi Le [a,b,c,#], Yiyuan Gan [b,#], Yihong Fu [b], Jiamin Liu [b], Wen Li [b], Xue Zou [d],

Zhixu Zhou [b,c,d], Zhenchao Wang [b,c,*], Guiping Ouyang [a,b,c,*] and Longjia Yan [b,c,*]

[a] State Key Laboratory Breeding Base of Green Pesticide and Agricultural Bioengineering, Key Laboratory of Green Pesticide and Agricultural Bioengineering, Ministry of Education, State-Local Joint Laboratory for Comprehensive Utilization of Biomass, Center for Research and Development of Fine Chemicals, Guizhou University, Guiyang 550025

[b] School of Pharmaceutical Sciences, Guizhou University, Guiyang 550025

[c] Guizhou Engineering Laboratory for Synthetic Drugs, Guiyang 550025

[d] Clinical Research Center, Affiliated Hospital of Guizhou Medical University, Guiyang, 550001

E-mail: oygp710@163.com (Guiping Ouyang); wzc.4884@163.com (Zhenchao Wang); ylj1089@163.com (Longjia Yan).

[#] Y. Le and Y. Gan contributed equally to this work.

Table of contents

MM/GBSA energy for compounds binding with EGFR …………………………...**2**

NMR and HRMS spectra of compounds **5a-p**…………………………………..…..**3-26**

Table S1 MM/GBSA energy for compounds binding with EGFR in active or inactive states.

Compd.	MM/GBSA Energy (kcal/mol)	
	EGFR in Active State	EGFR in Inactive State
**5a**	-32.95+3.24	-41.49+2.58
**5b**	-27.65+4.16	-43.69+3.27
**5c**	-25.17+2.41	-43.79+2.69
**5d**	-27.32+3.67	-42.81+2.18
**5e**	-27.26+4.51	-41.49+3.04
**5f**	-35.25+2.22	-42.93+2.58
**5g**	-22.43+3.1	-42.68+2.89
**5h**	-32.16+4.25	-41.88+2.55
**5i**	-32.81+2.84	-44.61+3.02
**5j**	-26.37+2.87	-45.29+2.58
**5k**	-24.84+4.8	-43.04+3.78
**5l**	-26.24+4.75	-43.67+2.4
**5m**	-35.12+2.23	-44.57+2.09
**5n**	-23.37+3.84	-40.3+3.75
**5o**	-27.06+3.88	-44.86+2.46
**5p**	-25.14+3.67	-45.61+2.63
**Gefitinib**	-45.68+2.52	-44.84+2.4

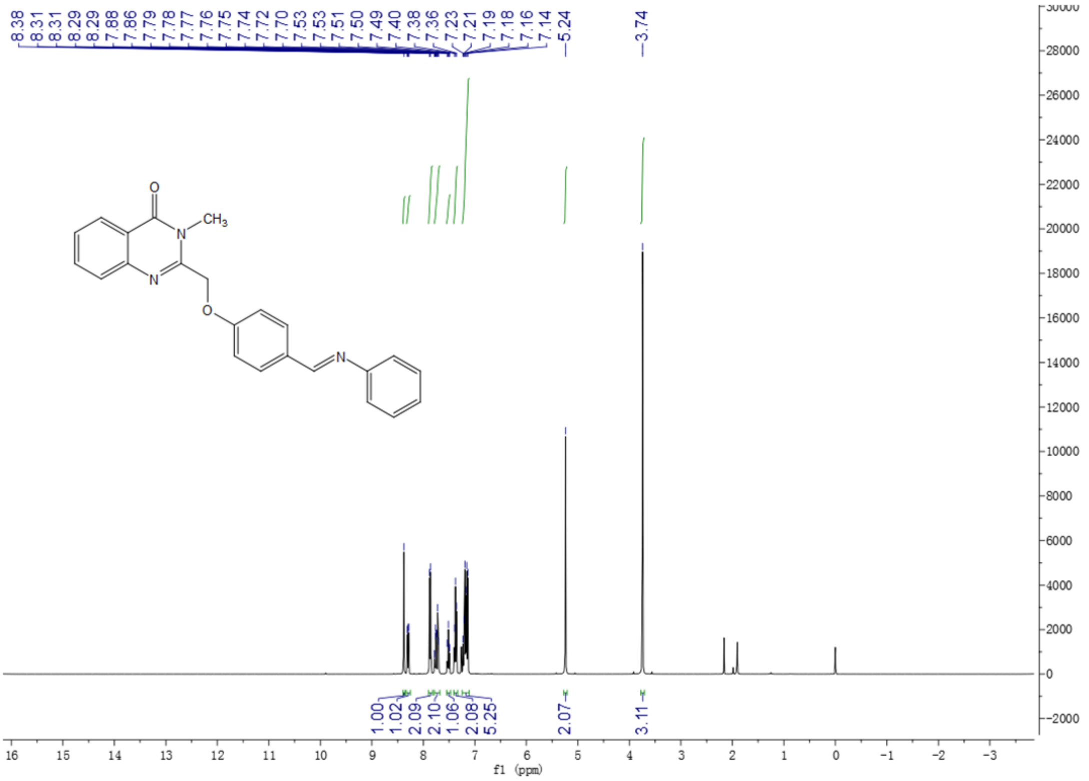

Fig. S1 ^{1}H NMR spectrogram (400 MHz, $CDCl_3$) of compound **5a**

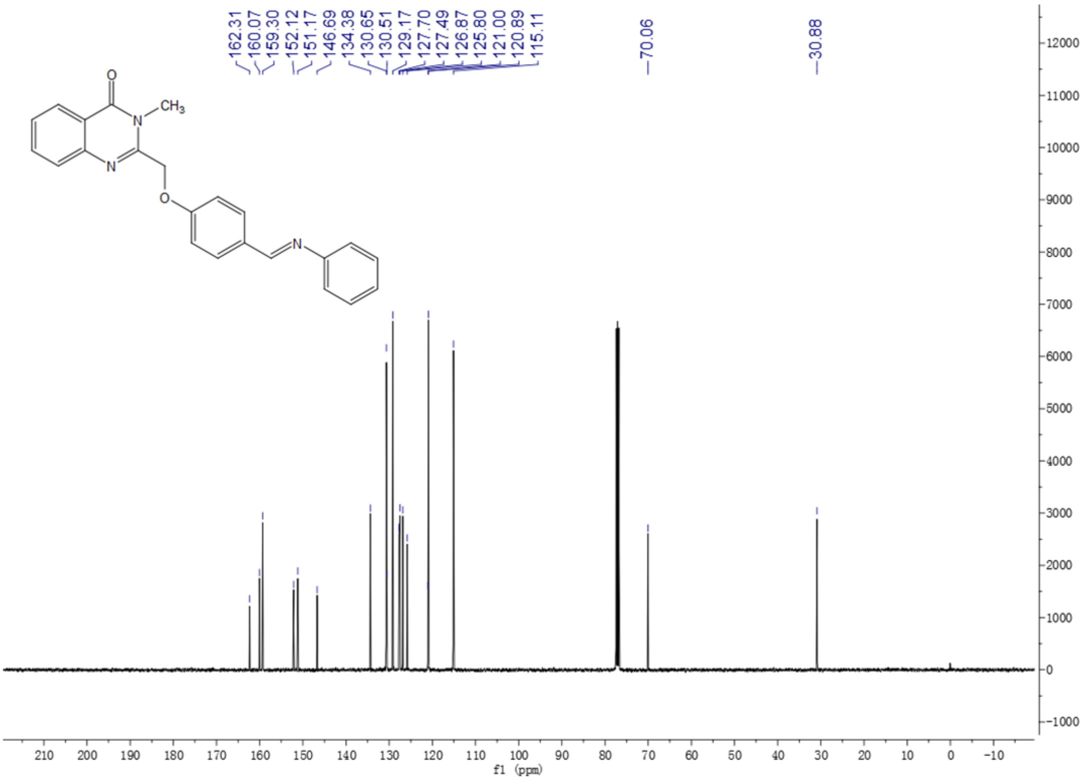

Fig.S2 ^{13}C NMR spectrogram (100 MHz, $CDCl_3$) of compound **5a**

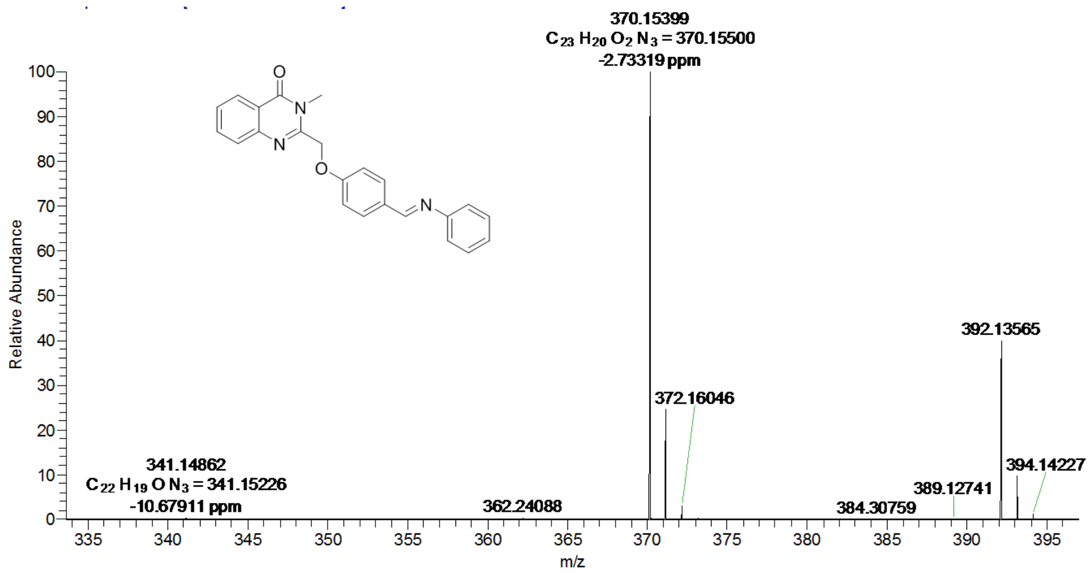

Fig.S3 HR-MS (ESI) spectrogram of compound 5a

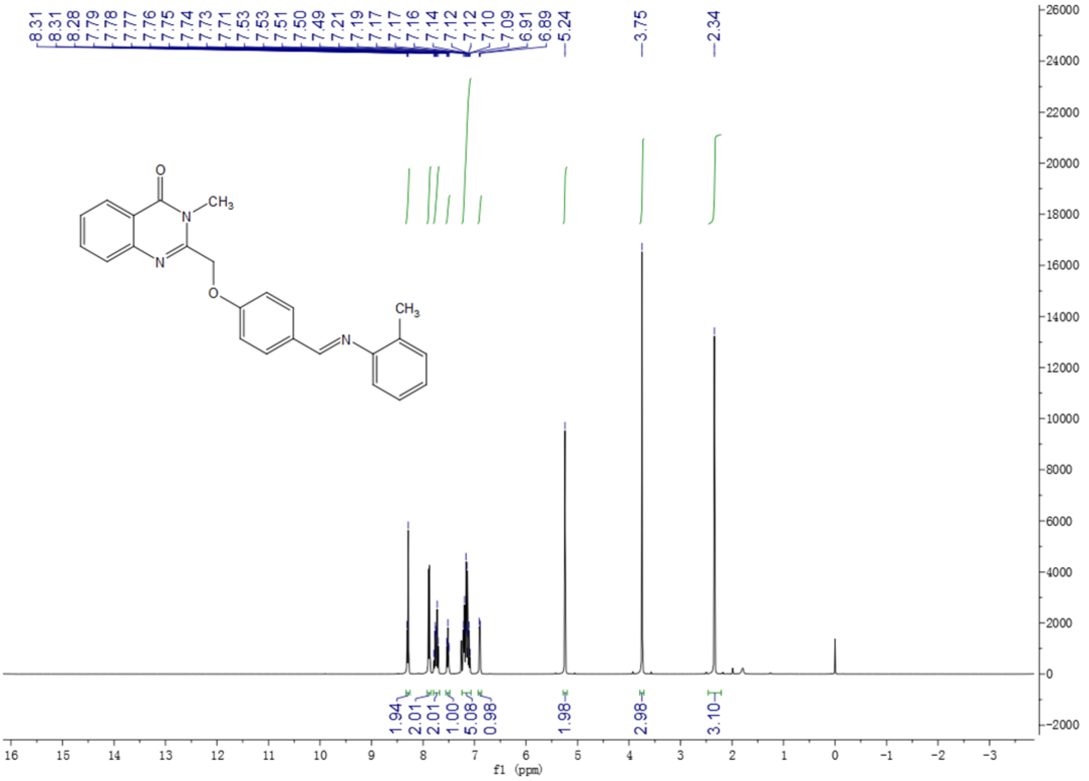

Fig.S4 ^{1}H NMR spectrogram (400 MHz, $CDCl_3$) of compound 5b

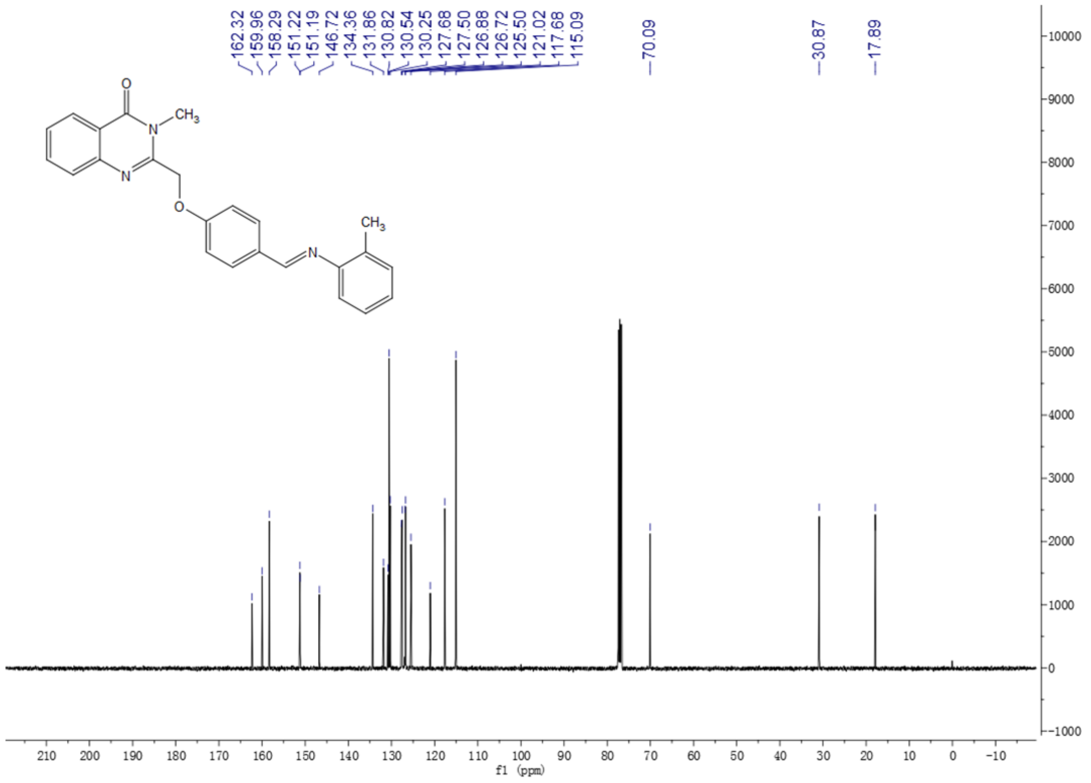

Fig.S5 ^{13}C NMR spectrogram (100 MHz, $CDCl_3$) of compound 5b

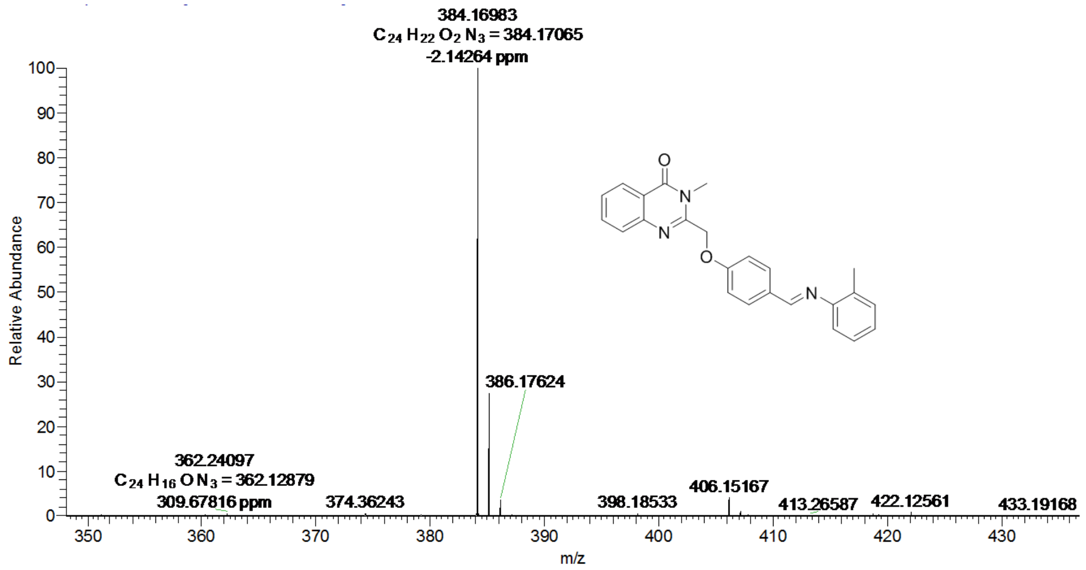

Fig.S6 HR-MS (ESI) spectrogram of compound 5b

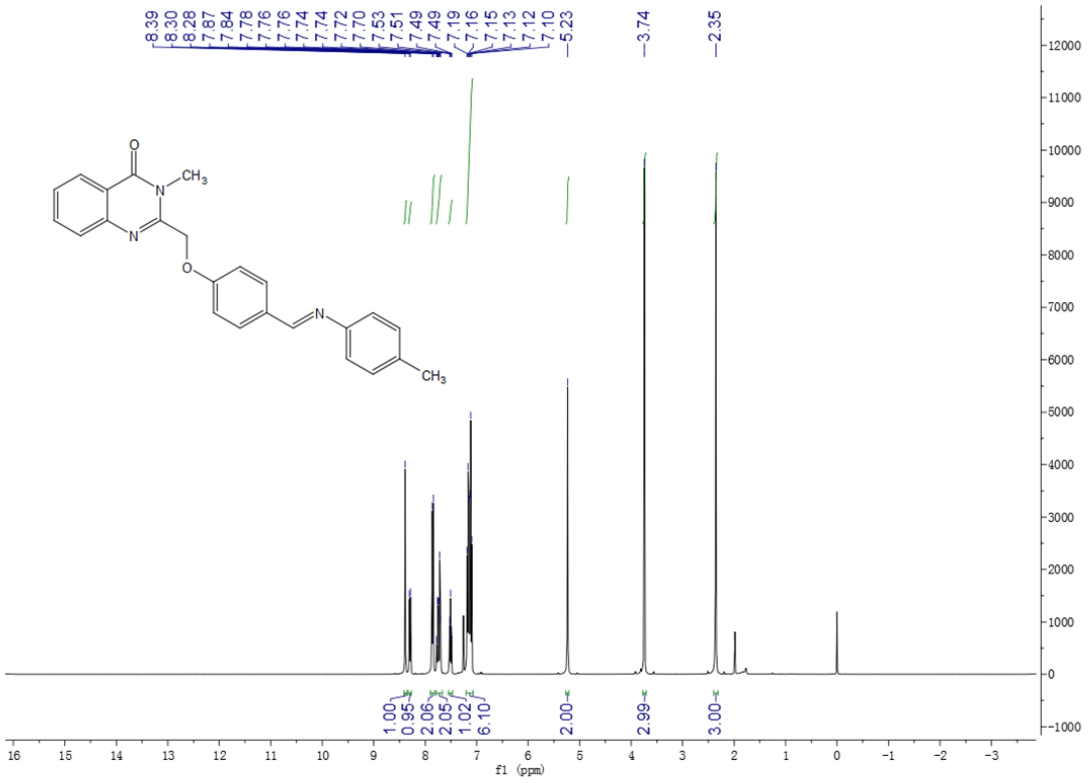

Fig.S7 ^{1}H NMR spectrogram (400 MHz, $CDCl_3$) of compound 5c

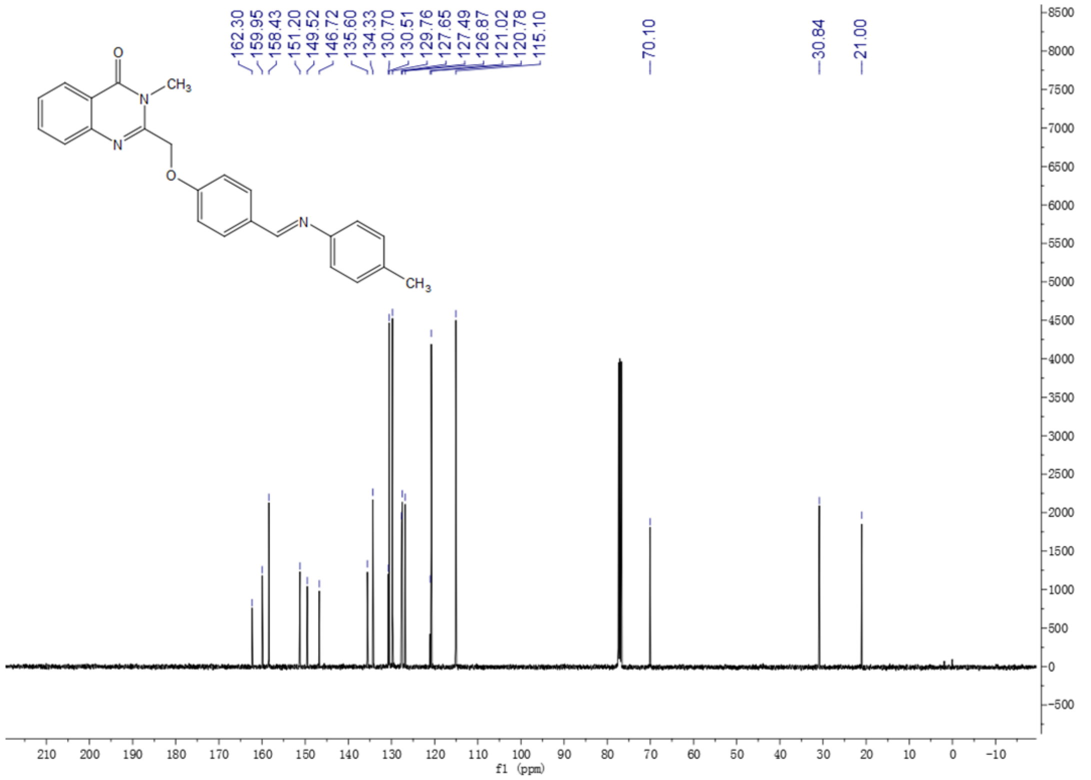

Fig.S8 ^{13}C NMR spectrogram (100 MHz, $CDCl_3$) of compound 5c

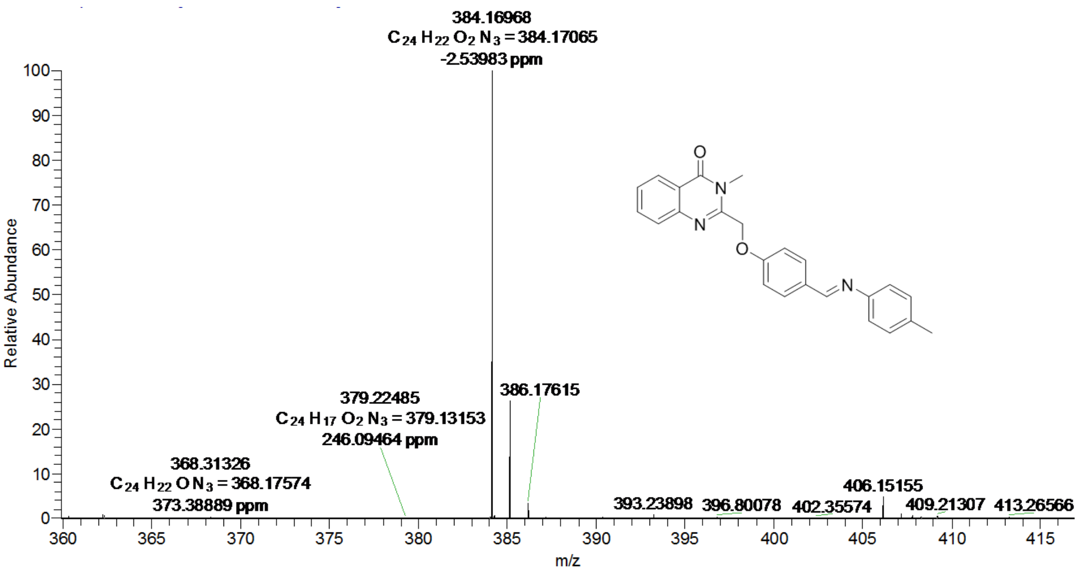

Fig.S9 HR-MS (ESI) spectrogram of compound 5c

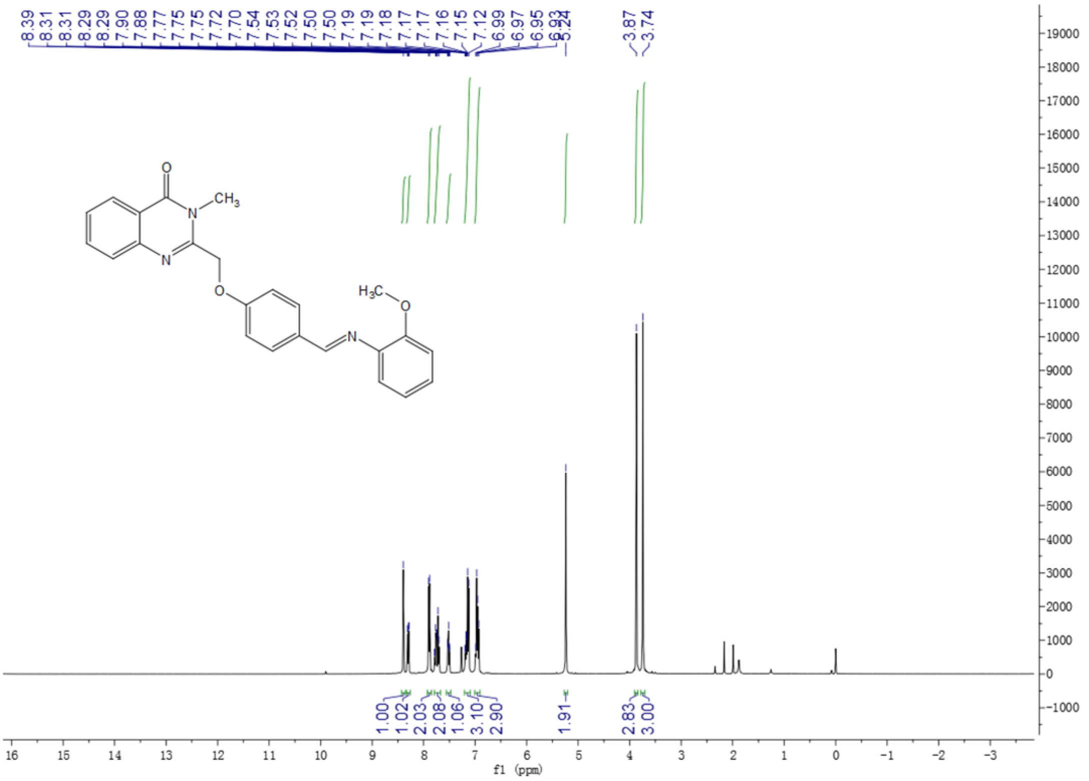

Fig.S10 ^{1}H NMR spectrogram (400 MHz, $CDCl_3$) of compound 5d

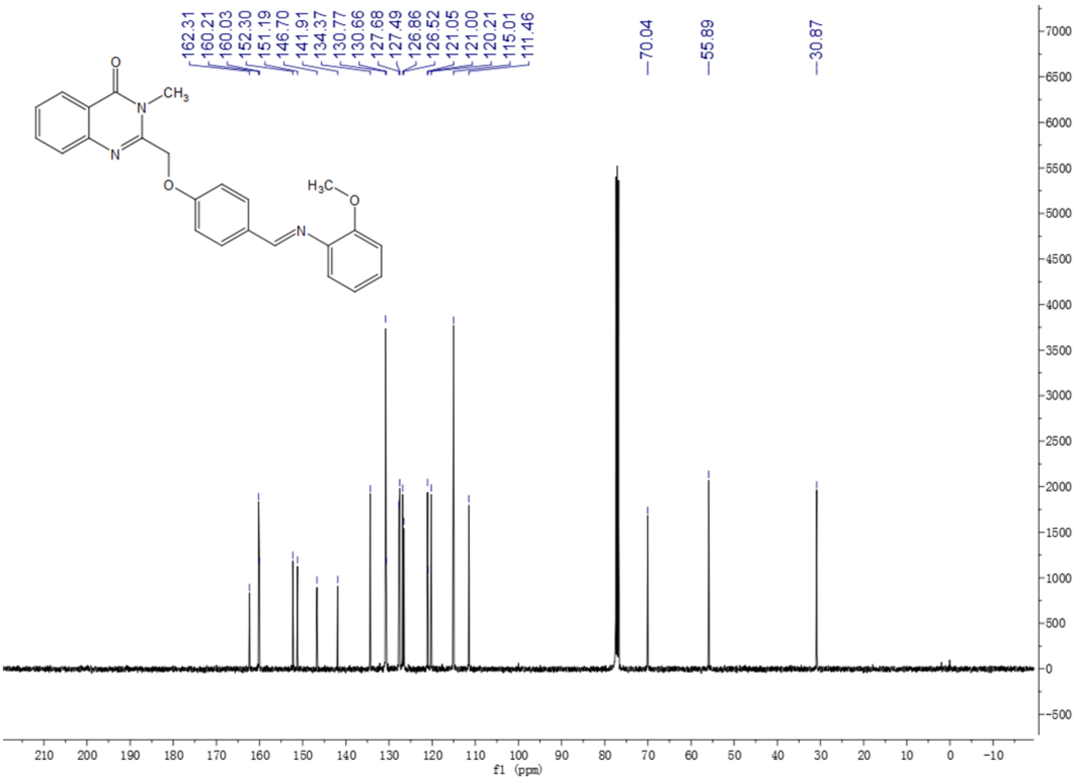

Fig.S11 [13]**C NMR spectrogram (100 MHz, CDCl**$_3$**) of compound 5d**

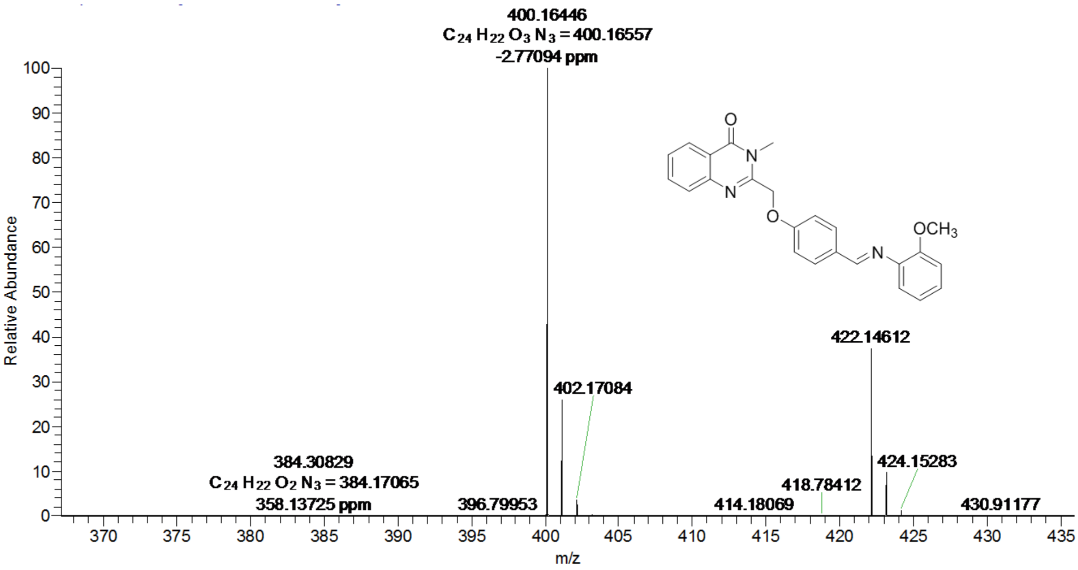

Fig.S12 HR-MS (ESI) spectrogram of compound 5d

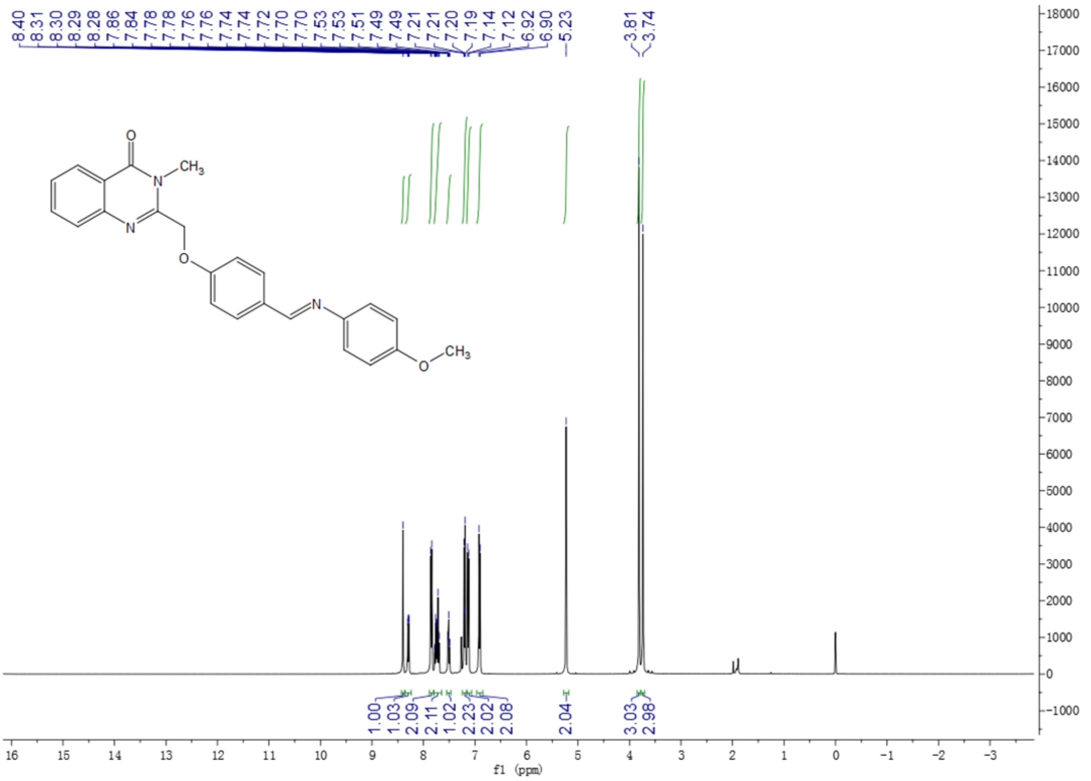

Fig.S13 ^{1}H **NMR spectrogram (400 MHz,** $CDCl_3$**) of compound 5e**

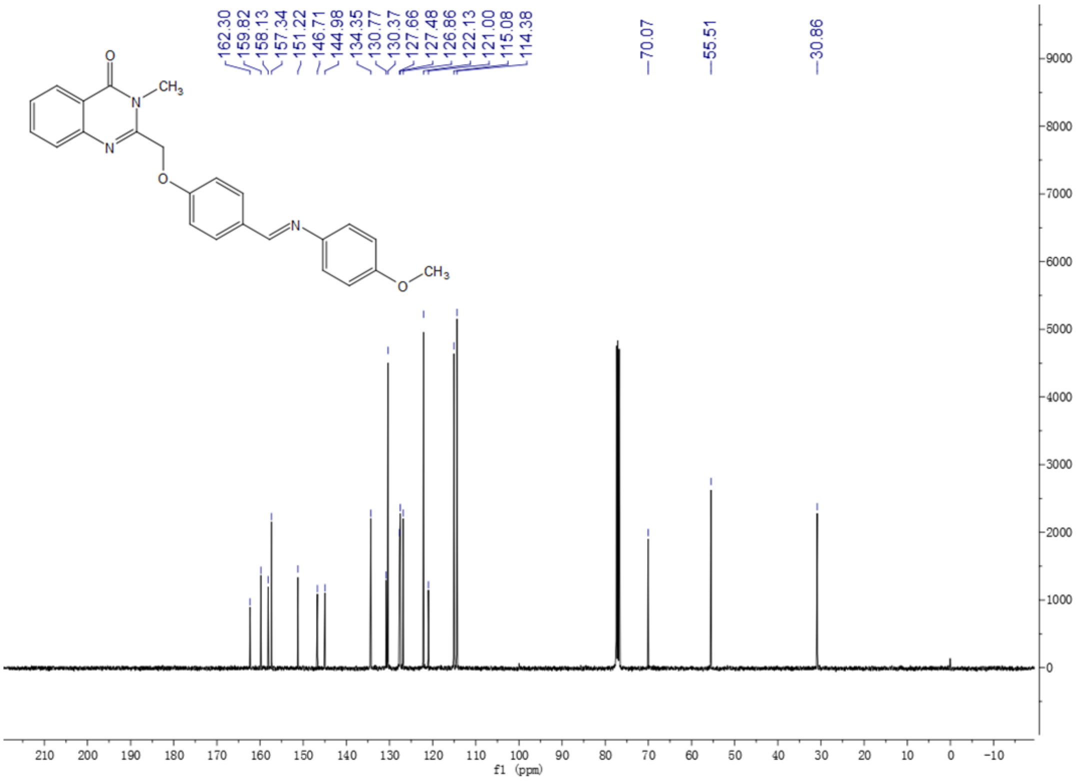

Fig.S14 ^{13}C **NMR spectrogram (100 MHz,** $CDCl_3$**) of compound 5e**

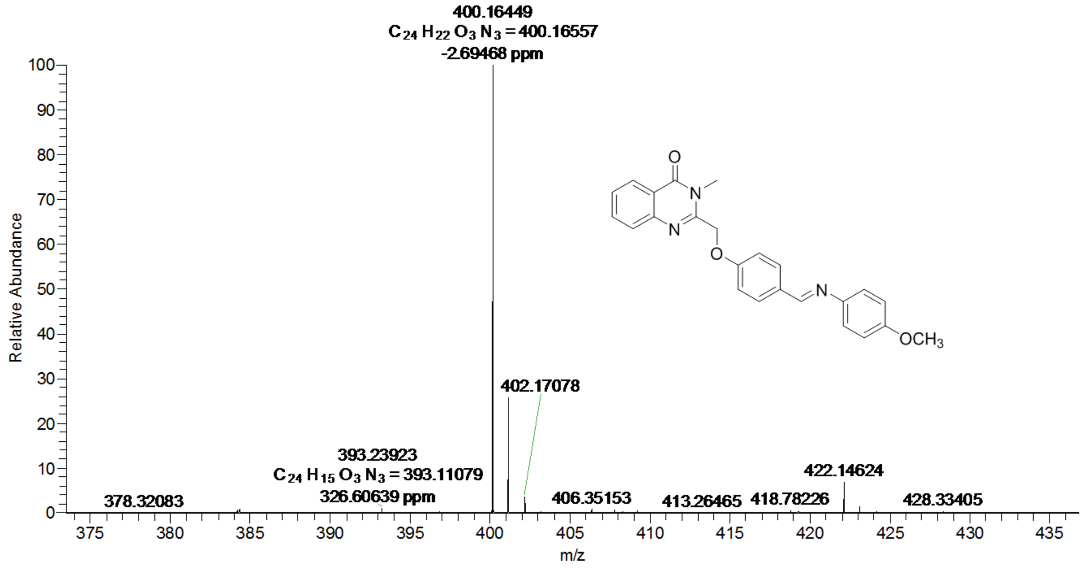

Fig.S15 HR-MS (ESI) spectrogram of compound 5e

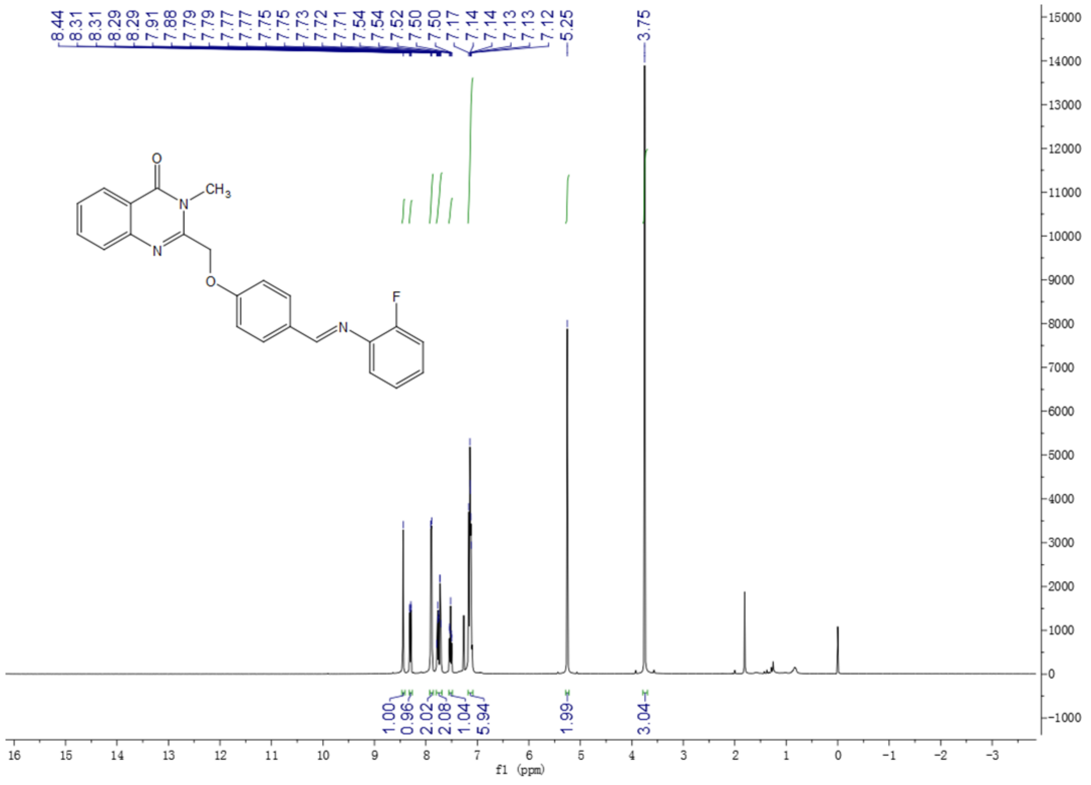

Fig.S16 ^{1}H NMR spectrogram (400 MHz, $CDCl_3$) of compound 5f

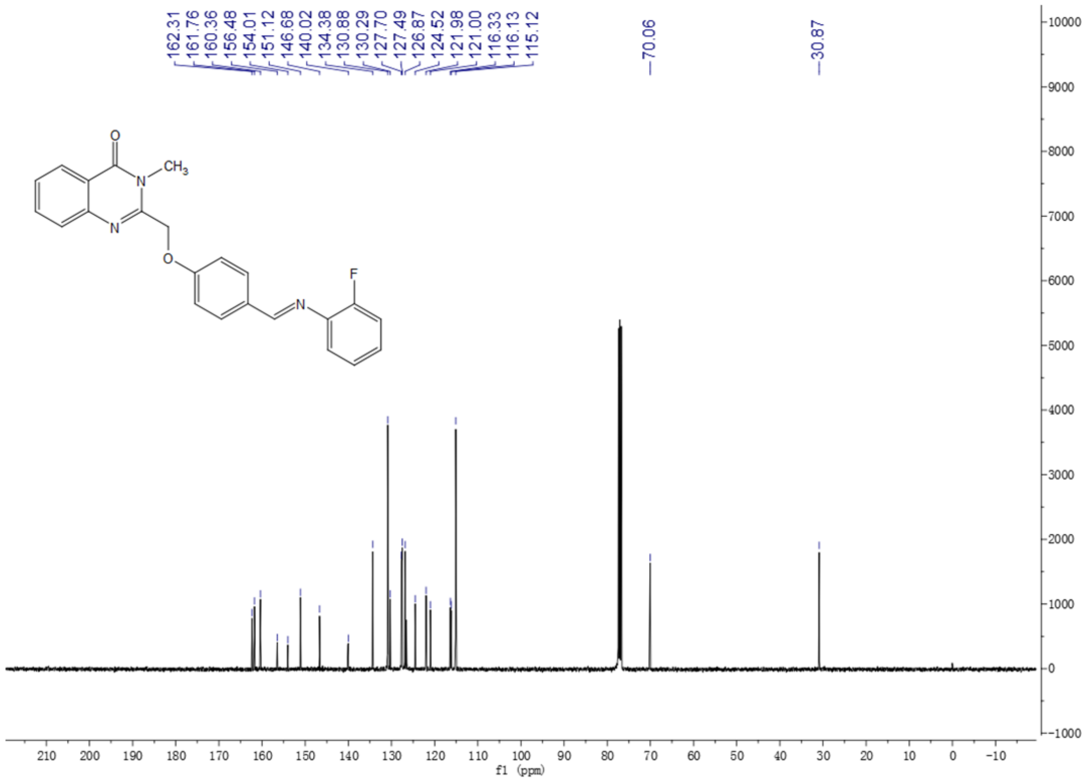

Fig.S17 ^{13}C NMR spectrogram (100 MHz, $CDCl_3$) of compound 5f

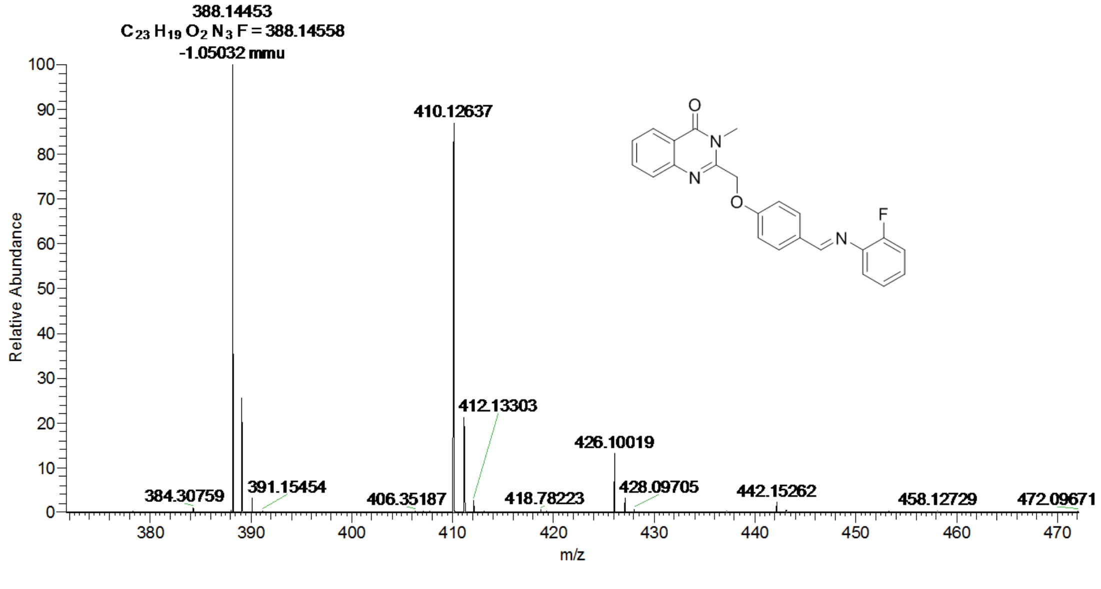

Fig.S18 HR-MS (ESI) spectrogram of compound 5f

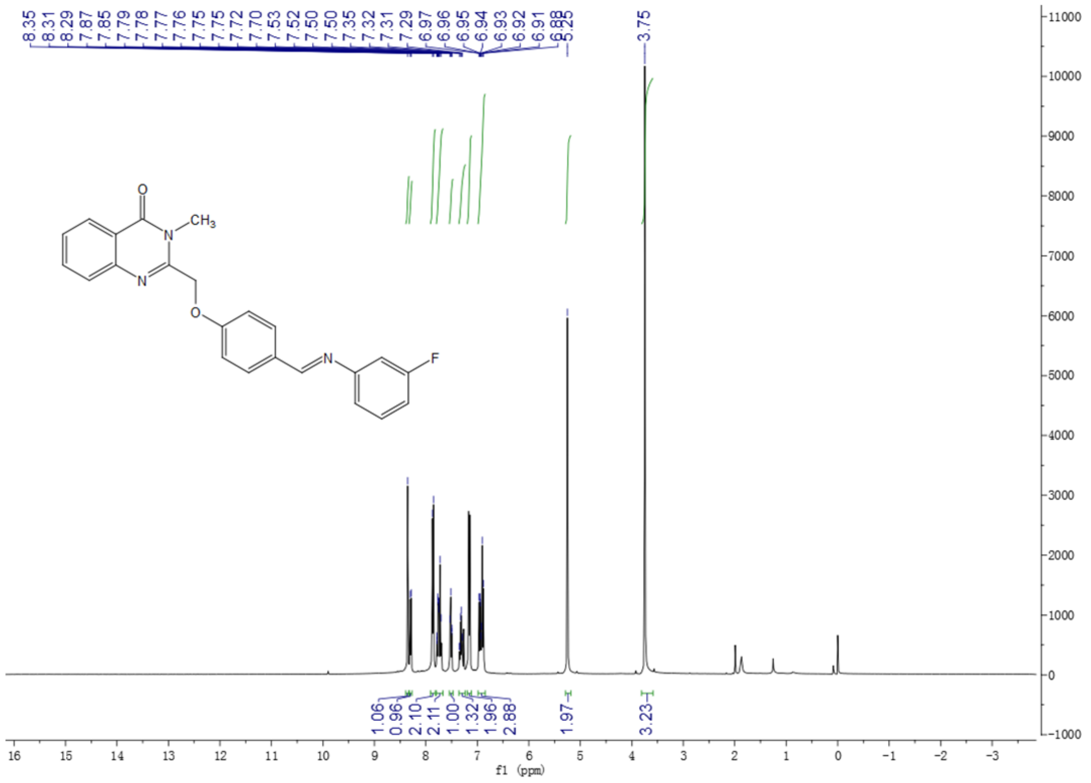

Fig.S19 ^{1}H NMR spectrogram (400 MHz, $CDCl_3$) of compound 5g

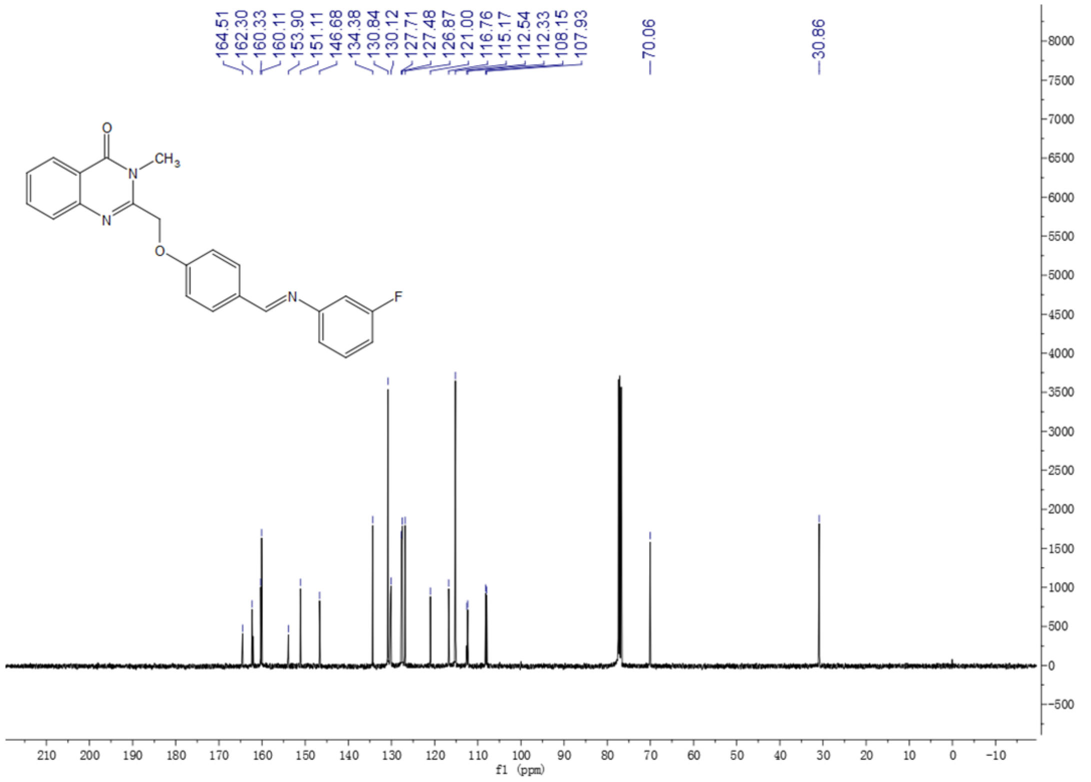

Fig.S20 ^{13}C NMR spectrogram (100 MHz, $CDCl_3$) of compound 5g

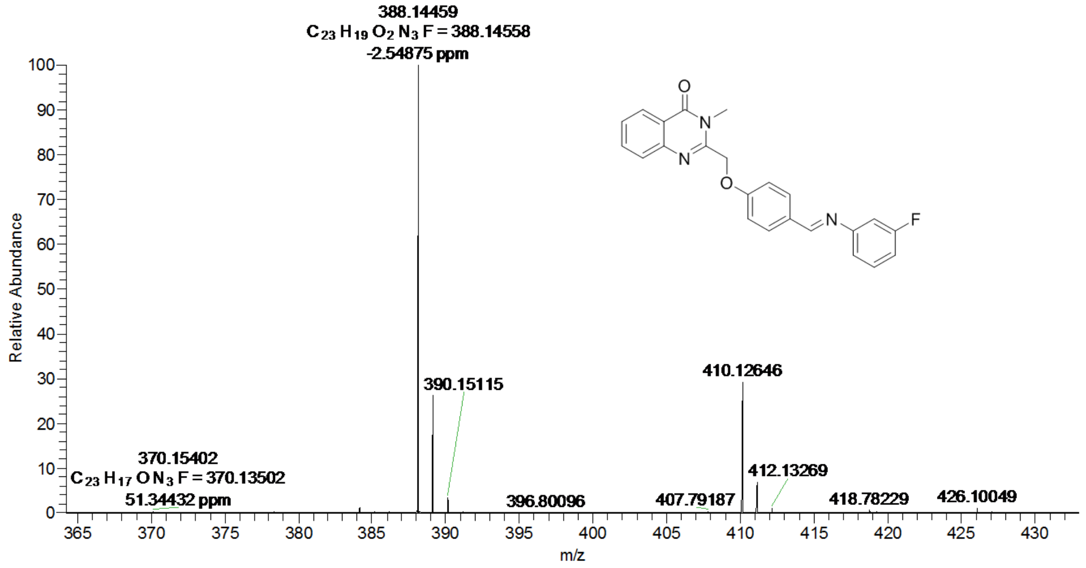

Fig.S21 HR-MS (ESI) spectrogram of compound 5g

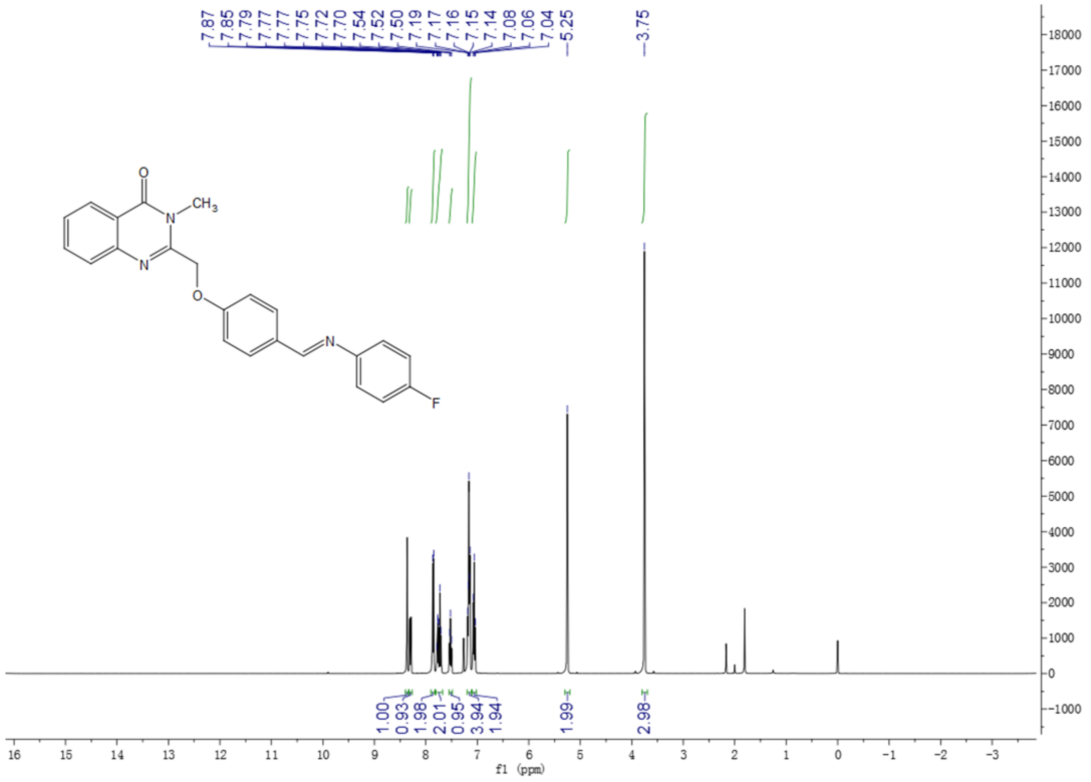

Fig.S22 ^{1}H NMR spectrogram (400 MHz, $CDCl_3$) of compound 5h

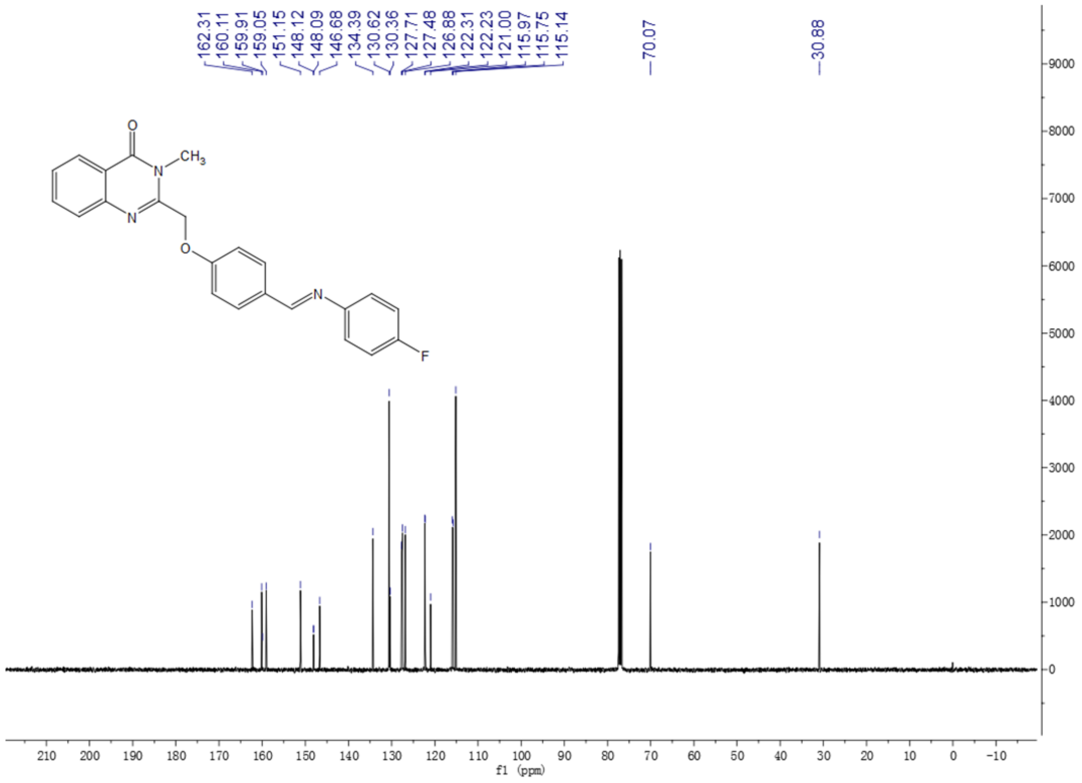

Fig.S23 ^{13}C NMR spectrogram (100 MHz, $CDCl_3$) of compound 5h

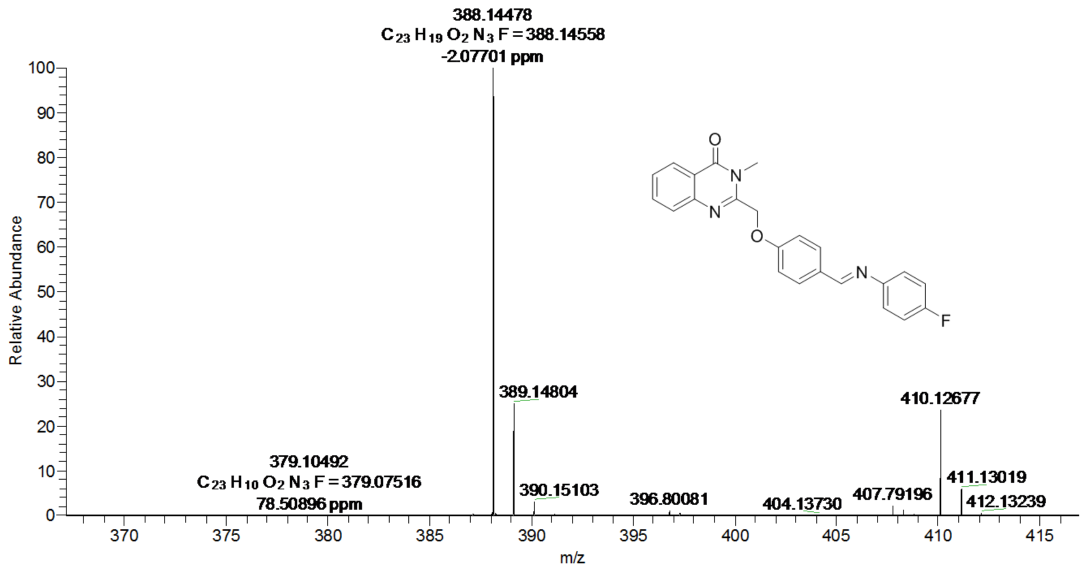

Fig.S24 HR-MS (ESI) spectrogram of compound 5h

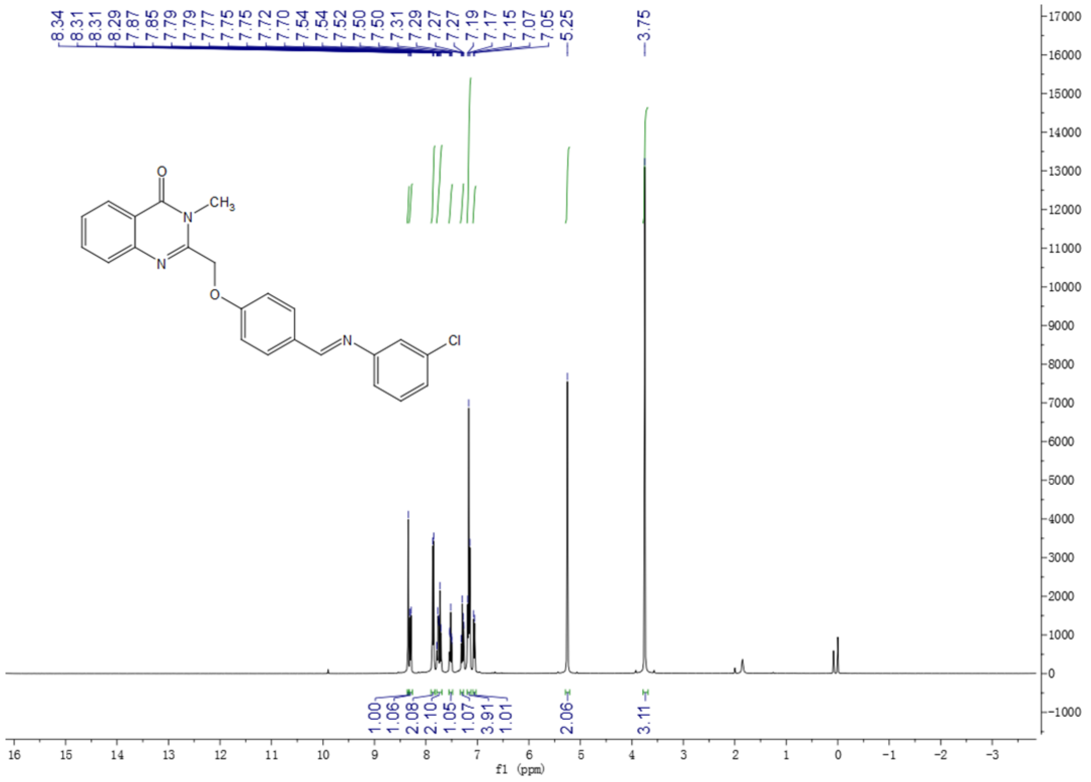

Fig.S25 ^{1}H NMR spectrogram (400 MHz, $CDCl_3$) of compound 5i

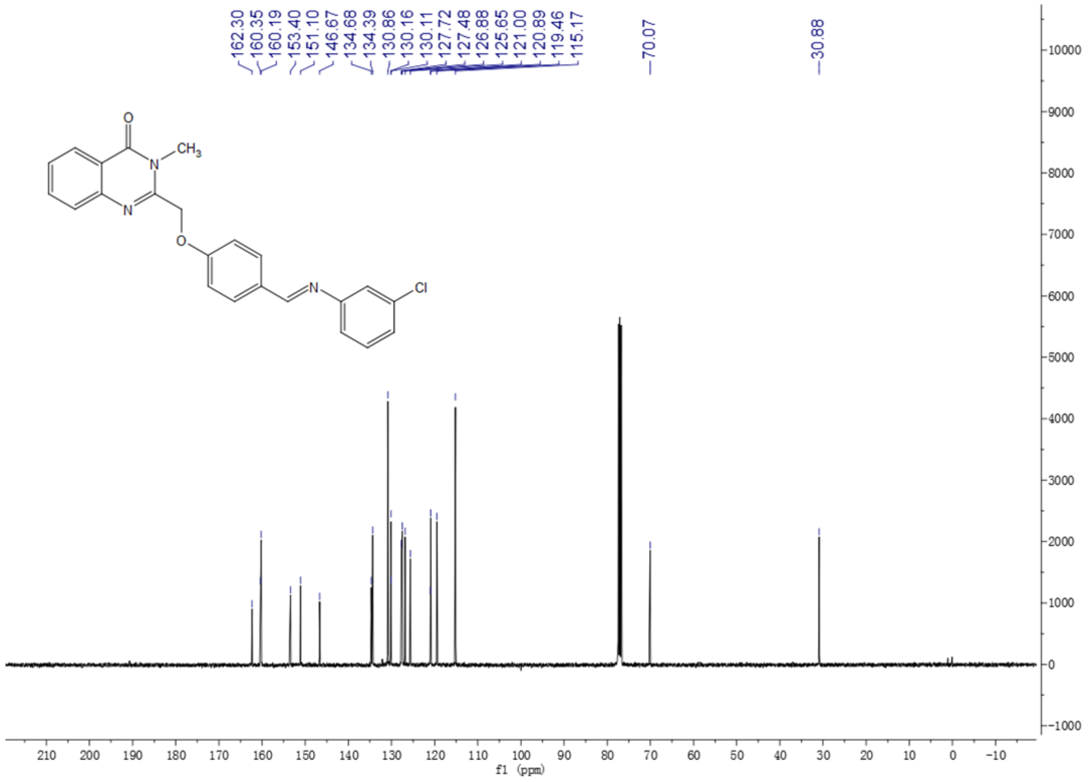

Fig.S26 ^{13}C NMR spectrogram (100 MHz, $CDCl_3$) of compound 5i

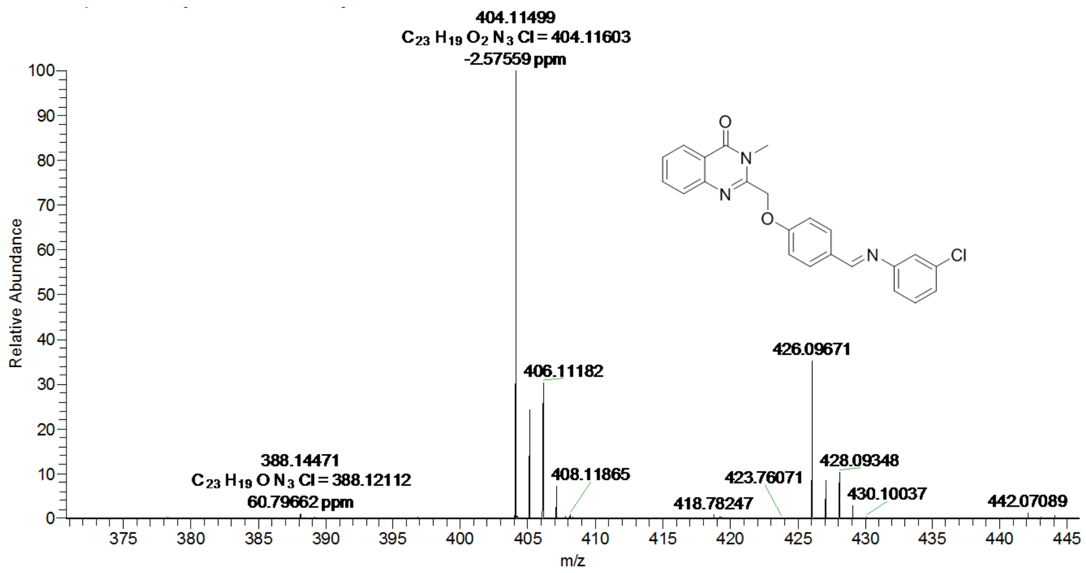

Fig.S27 HR-MS (ESI) spectrogram of compound 5i

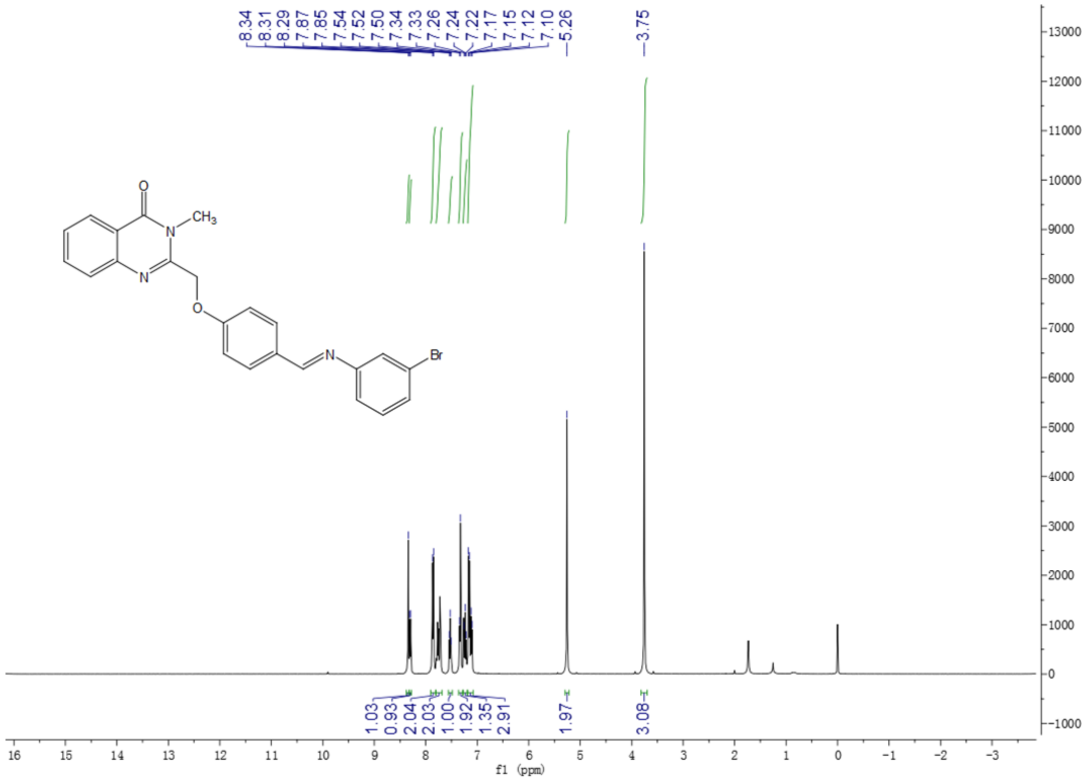

Fig.S28 ^{1}H NMR spectrogram (400 MHz, $CDCl_3$) of compound 5j

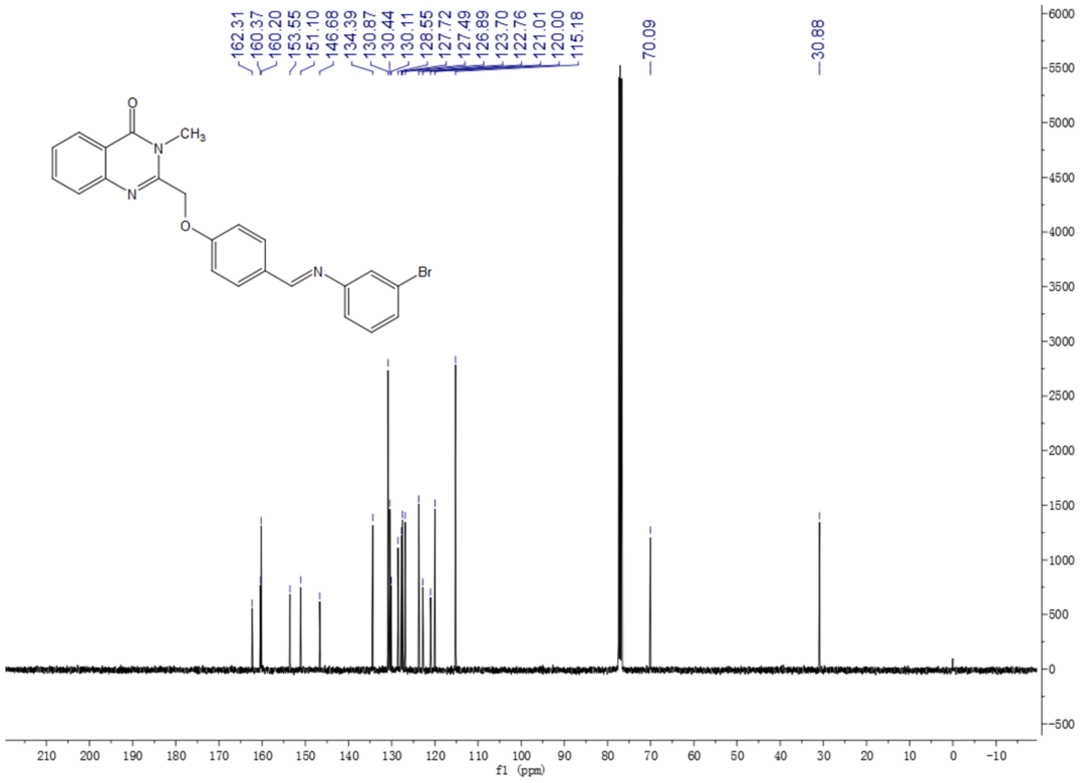

Fig.S29 ^{13}C NMR spectrogram (100 MHz, $CDCl_3$) of compound 5j

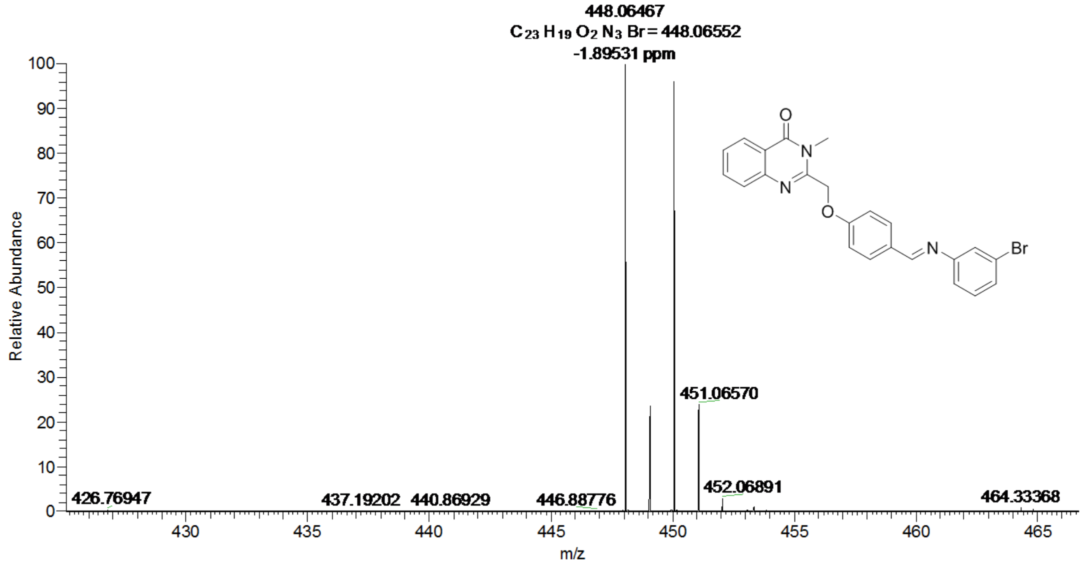

Fig.S30 HR-MS (ESI) spectrogram of compound 5j

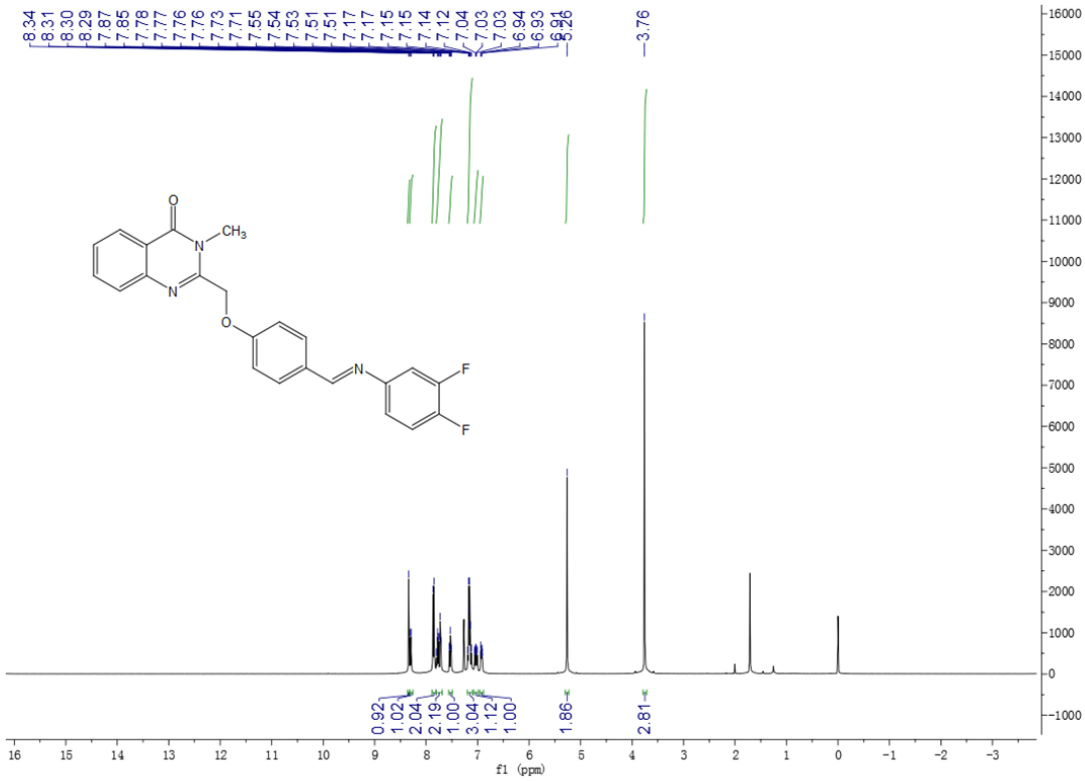

Fig.S31 ^{1}H NMR spectrogram (400 MHz, $CDCl_3$) of compound 5k

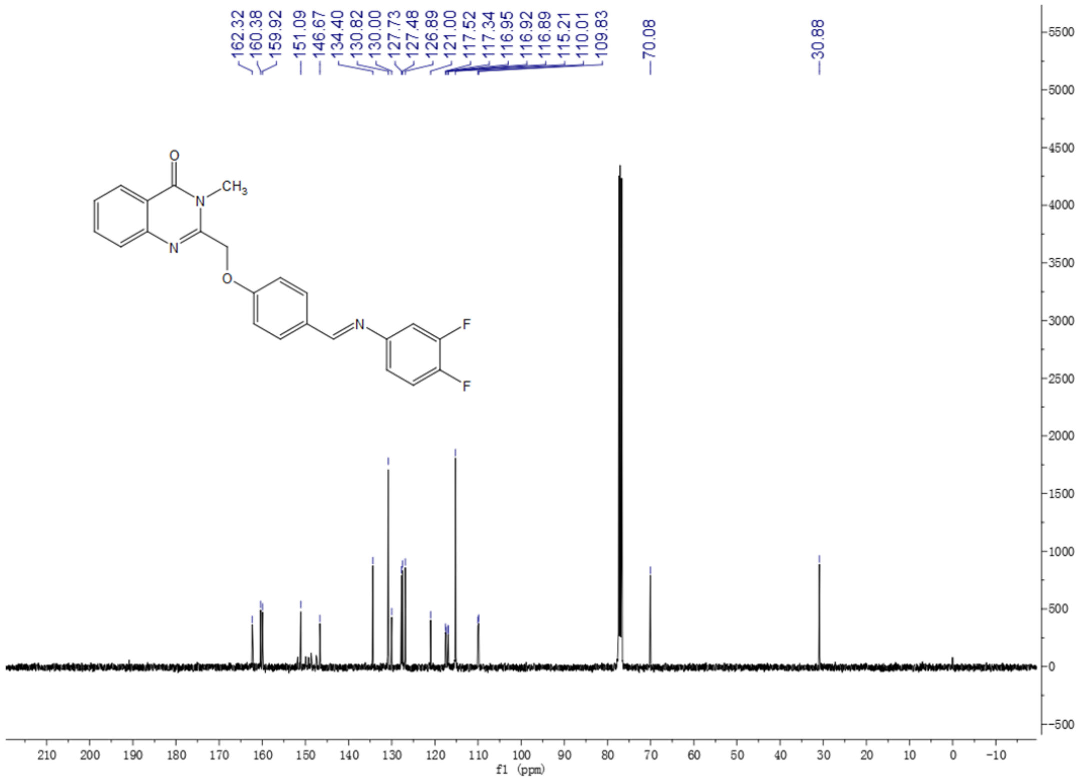

Fig.S32 ^{13}C NMR spectrogram (100 MHz, $CDCl_3$) of compound 5k

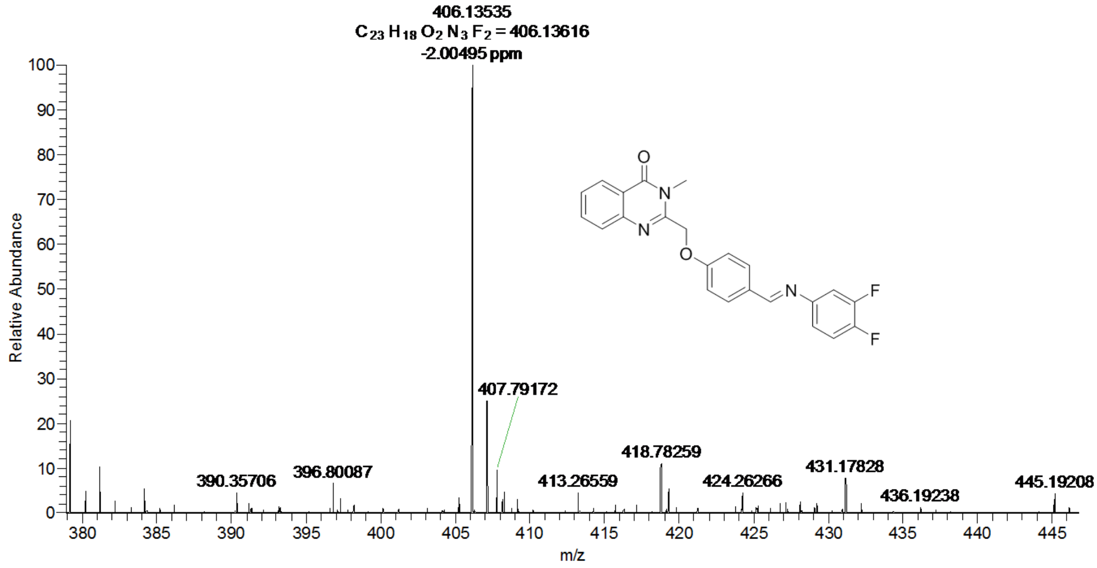

Fig.S33 HR-MS (ESI) spectrogram of compound 5k

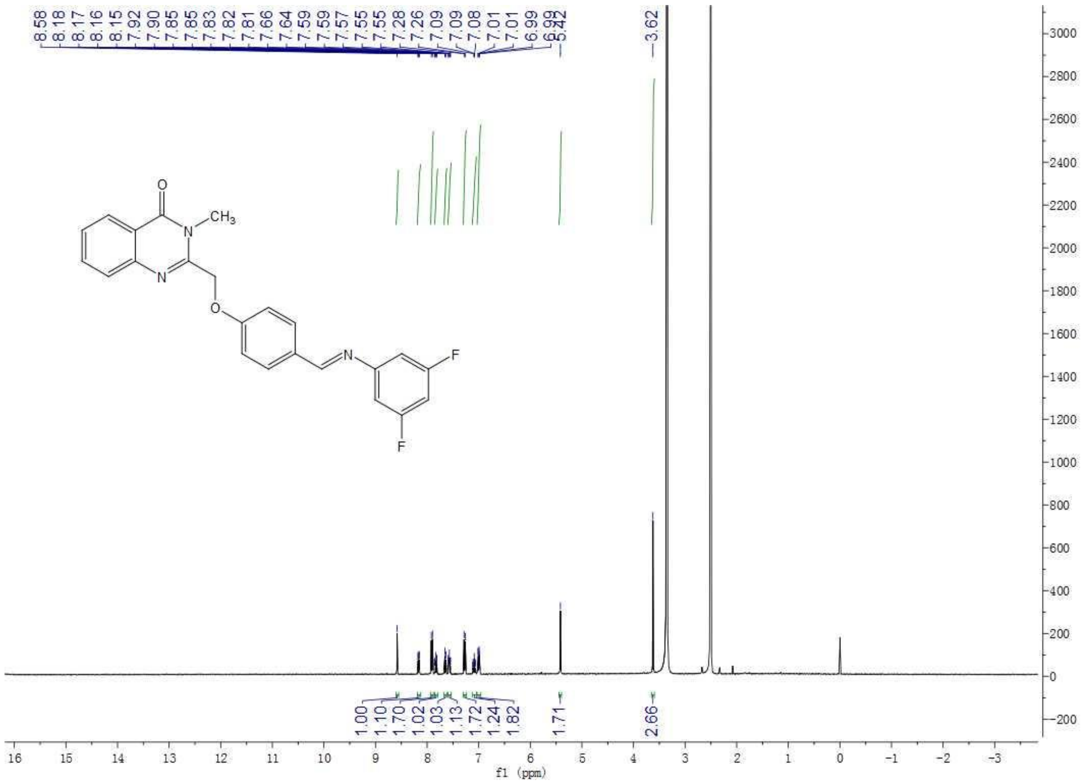

Fig.S34 ^{1}H NMR spectrogram (400 MHz, DMSO) of compound 5l

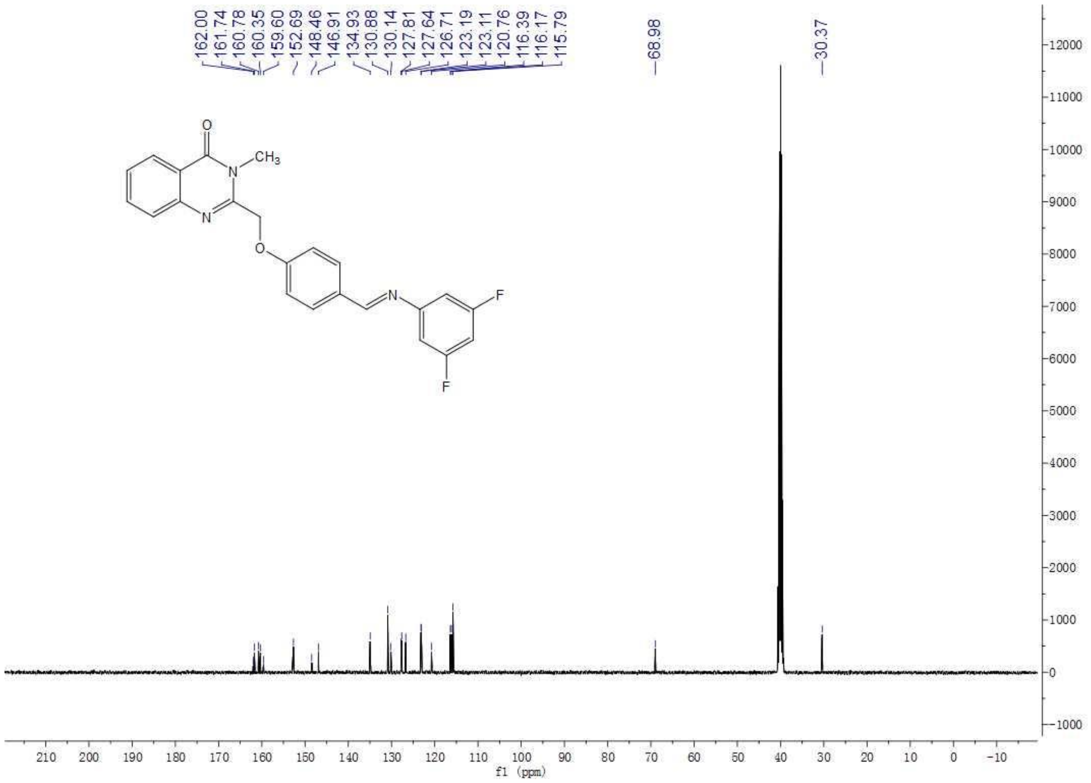

Fig.S35 ^{13}C NMR spectrogram (100 MHz, DMSO) of compound 5l

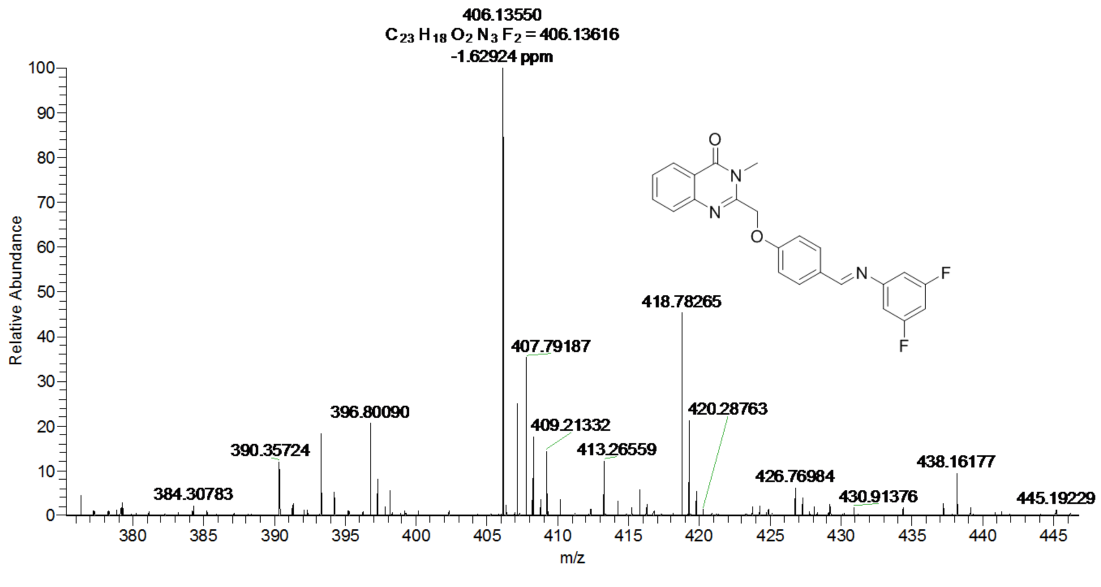

Fig.S36 HR-MS (ESI) spectrogram of compound 5l

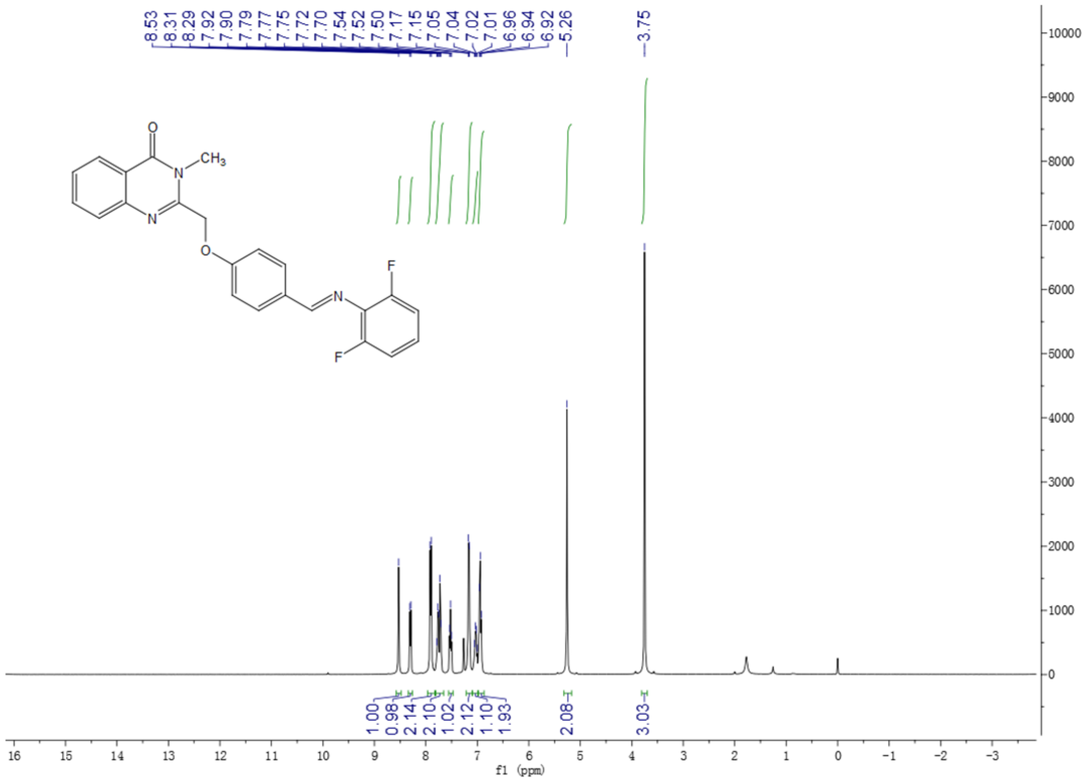

Fig.S37 ^{1}H NMR spectrogram (400 MHz, $CDCl_3$) of compound 5m

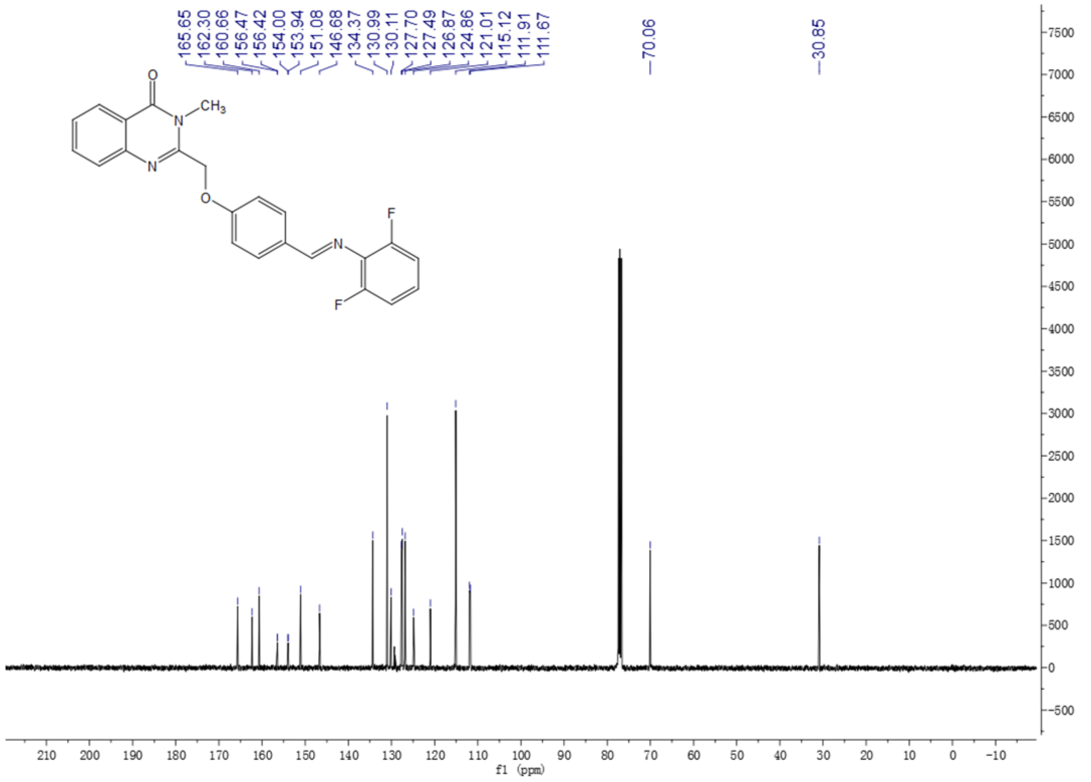

Fig.S38 ^{13}C NMR spectrogram (100 MHz, $CDCl_3$) of compound 5m

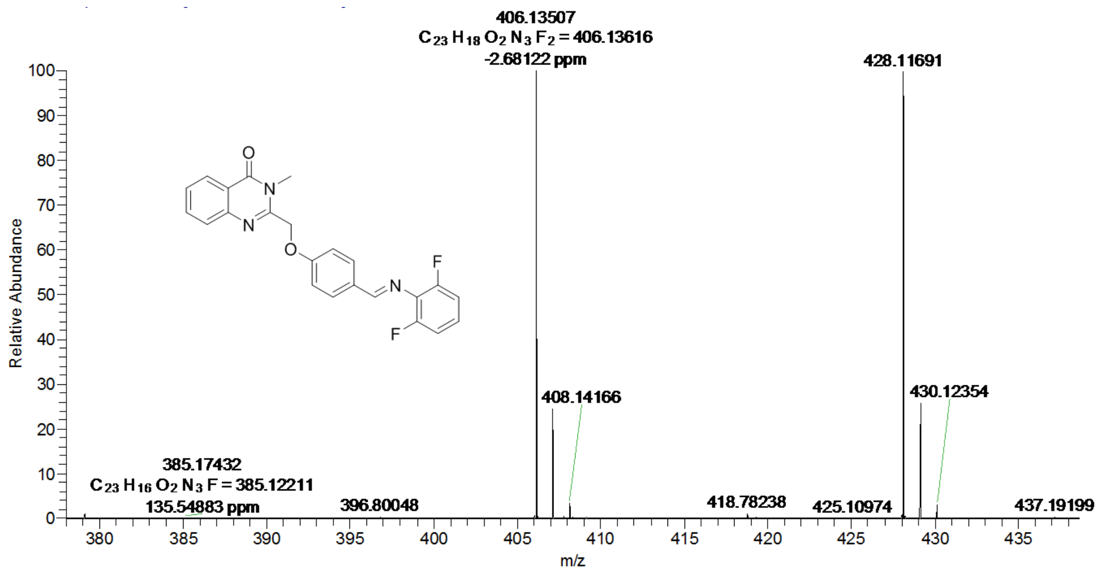

Fig.S39 HR-MS (ESI) spectrogram of compound 5m

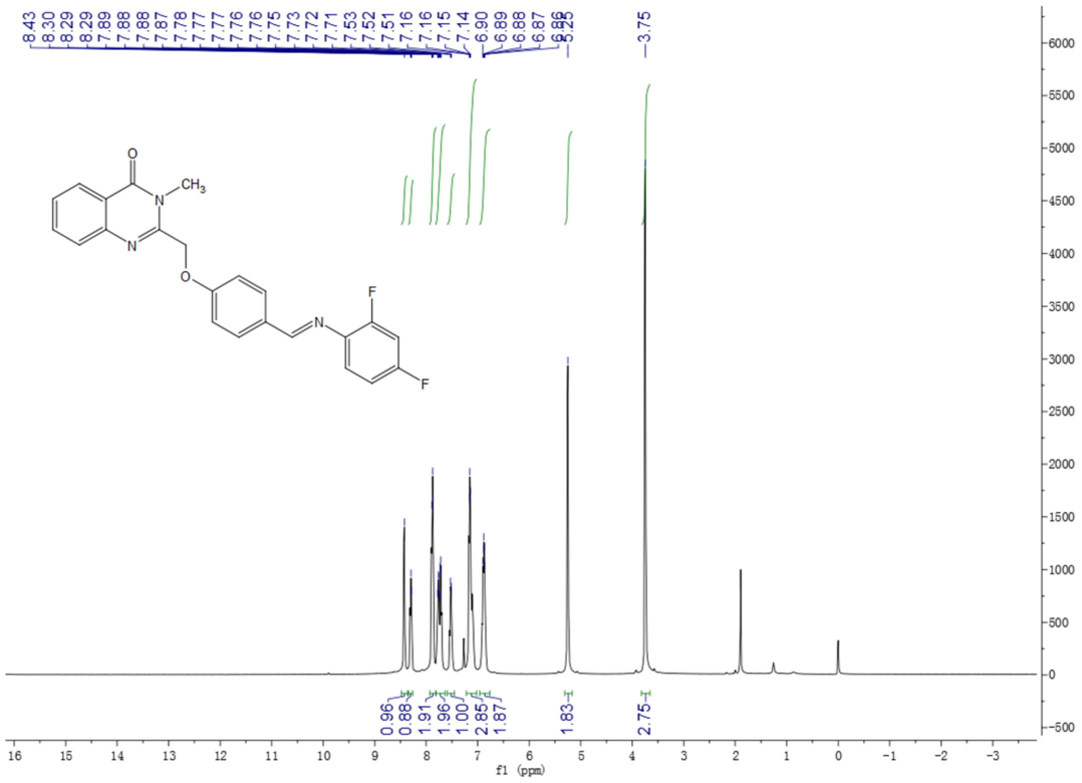

Fig.S40 ^{1}H NMR spectrogram (400 MHz, $CDCl_3$) of compound 5n

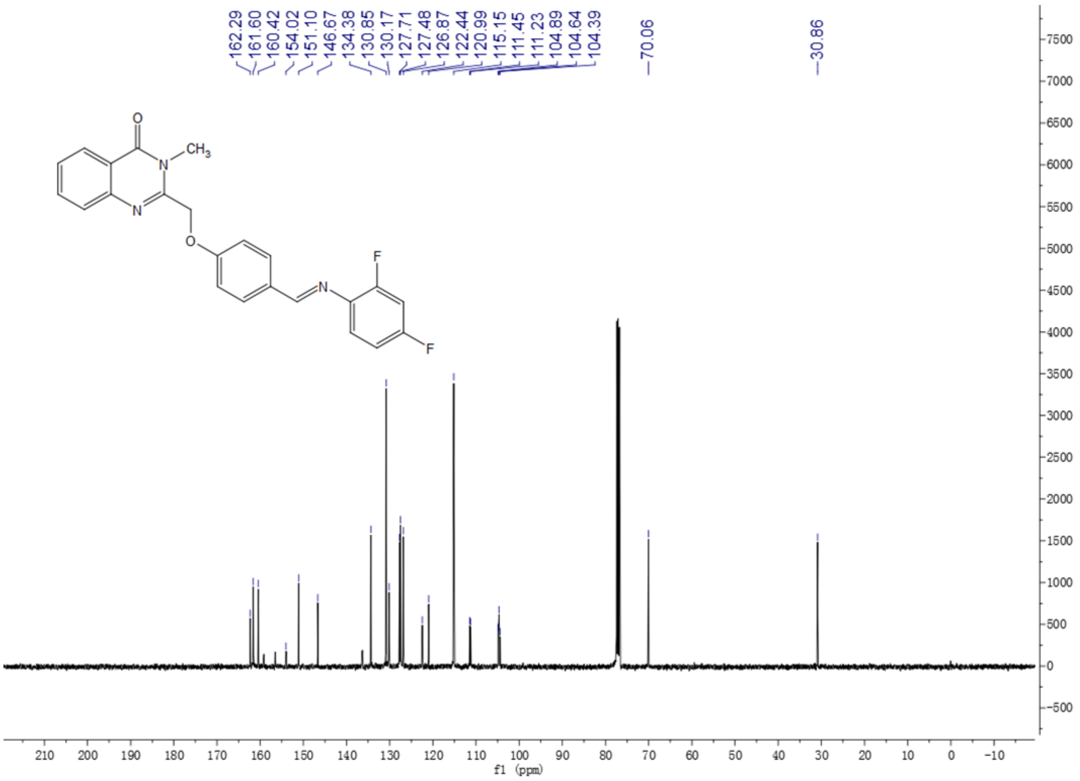

Fig.S41 ^{13}C NMR spectrogram (100 MHz, $CDCl_3$) of compound 5n

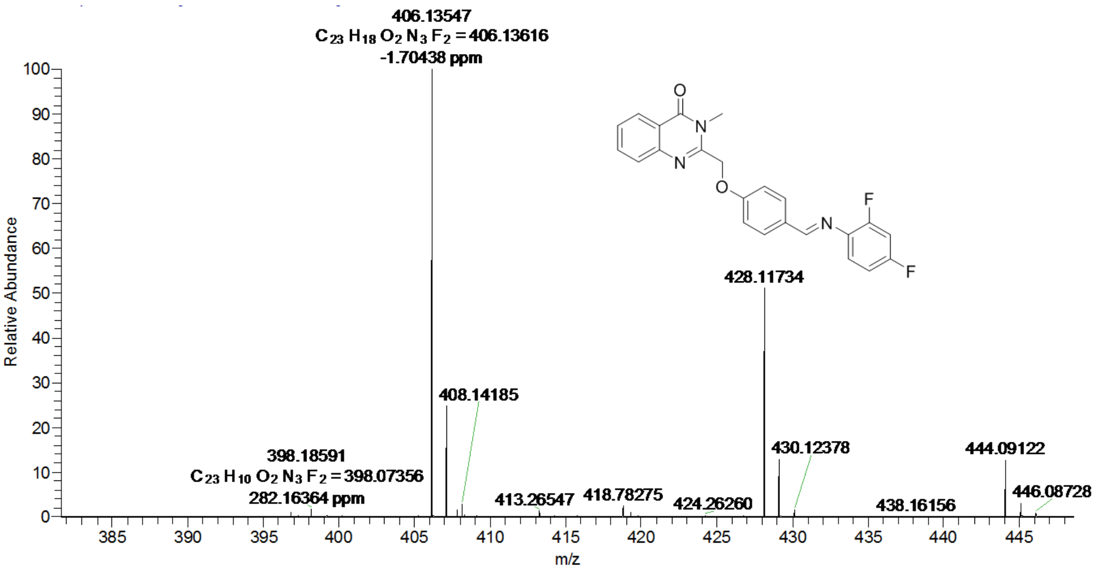

Fig.S42 HR-MS (ESI) spectrogram of compound 5n

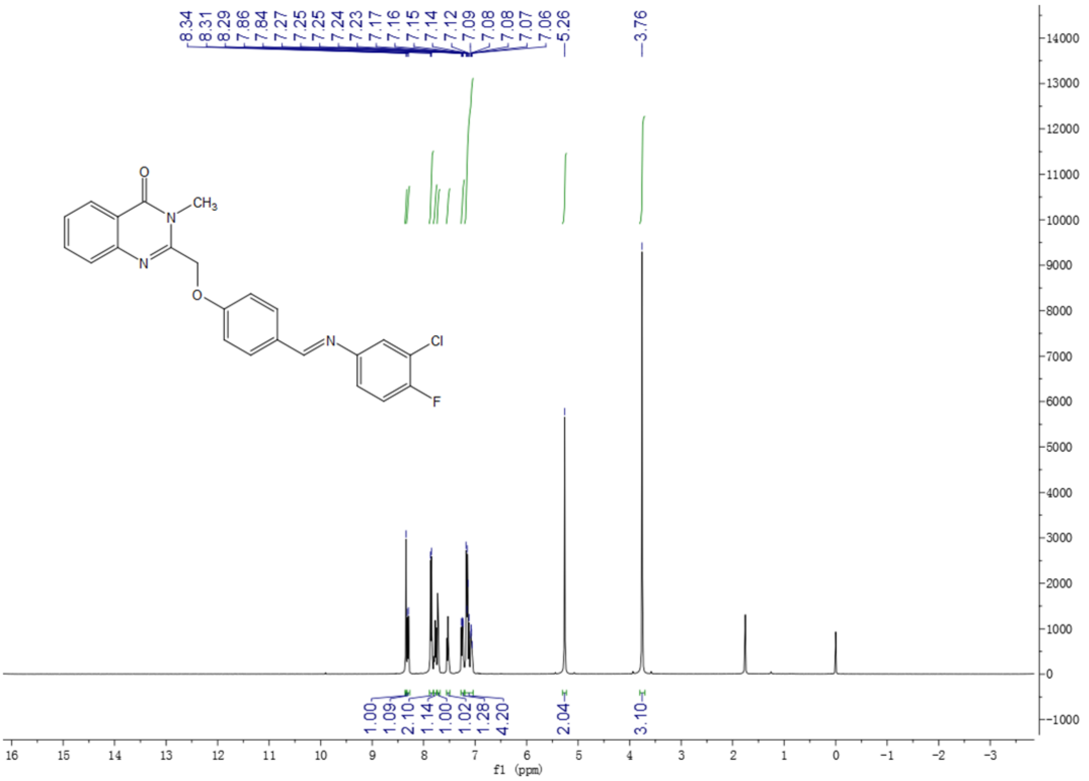

Fig.S43 ^{1}H NMR spectrogram (400 MHz, $CDCl_3$) of compound 5o

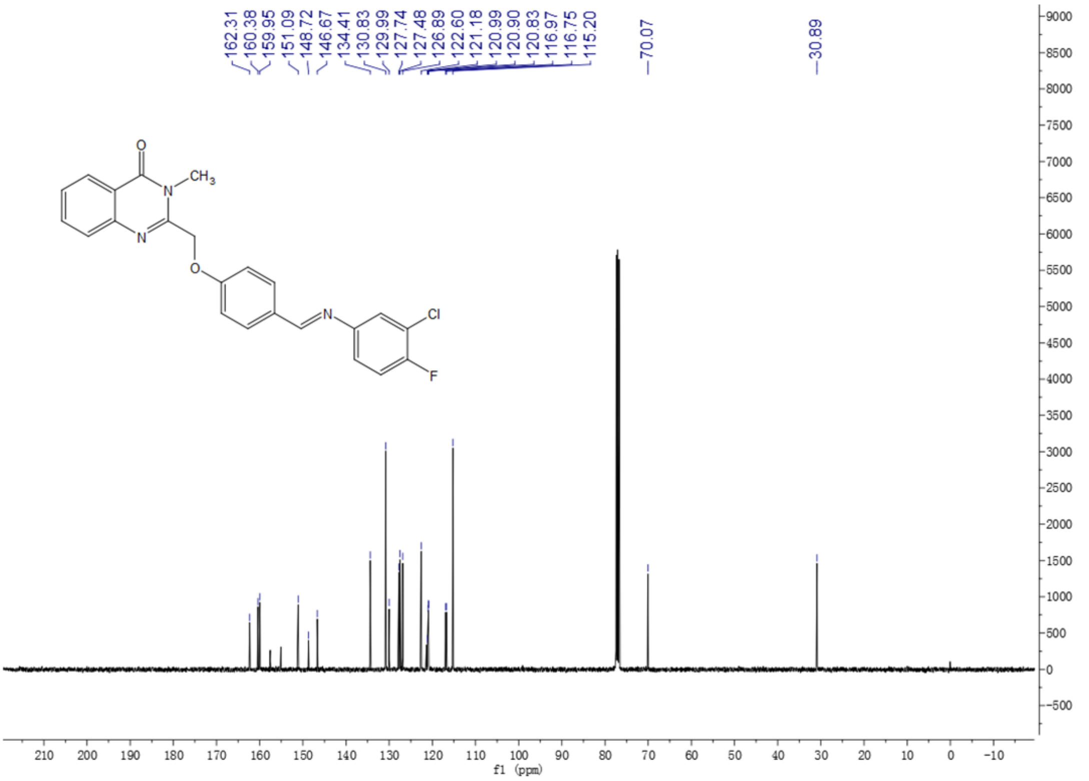

Fig.S44 ^{13}C NMR spectrogram (100 MHz, $CDCl_3$) of compound 5o

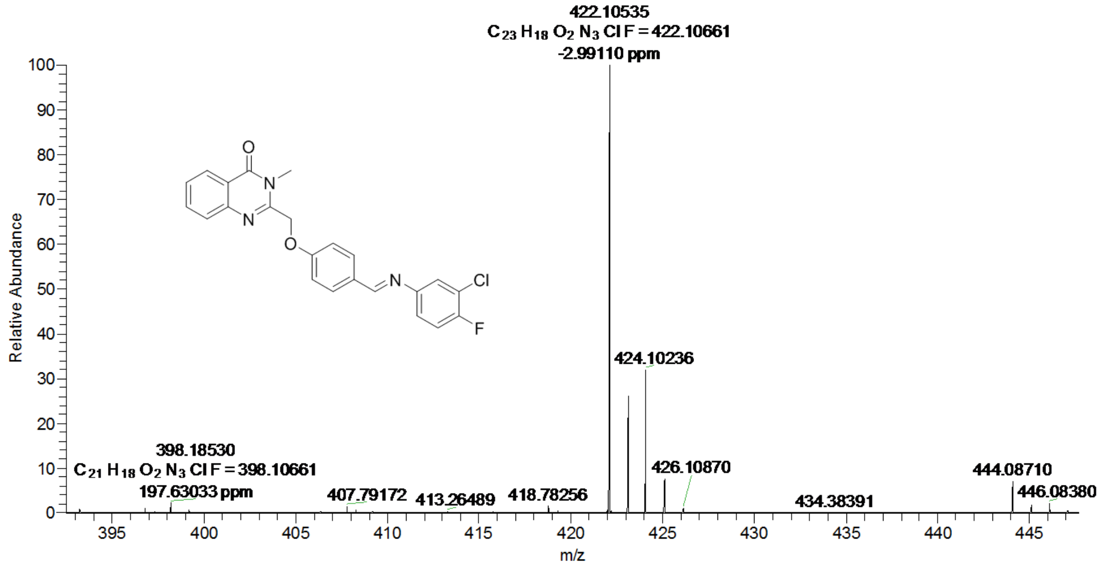

Fig.S45 HR-MS (ESI) spectrogram of compound 5o

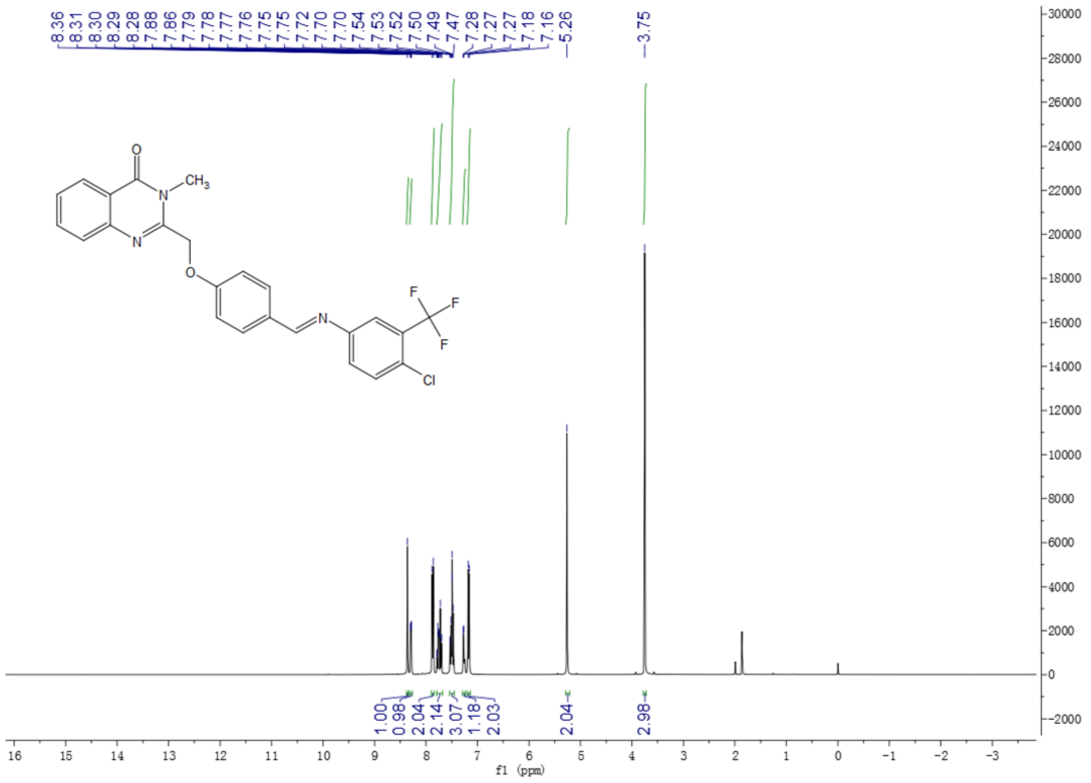

Fig.S46 ^{1}H NMR spectrogram (400 MHz, $CDCl_3$) of compound 5p

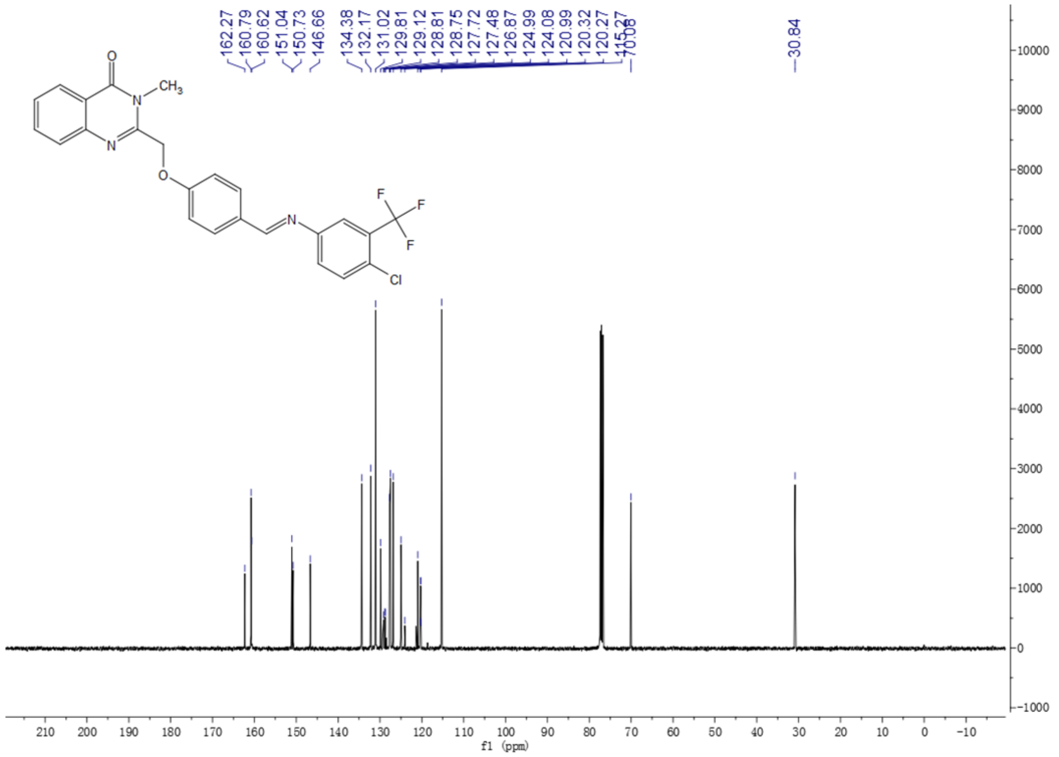

Fig.S47 ^{13}C NMR spectrogram (100 MHz, $CDCl_3$) of compound 5p

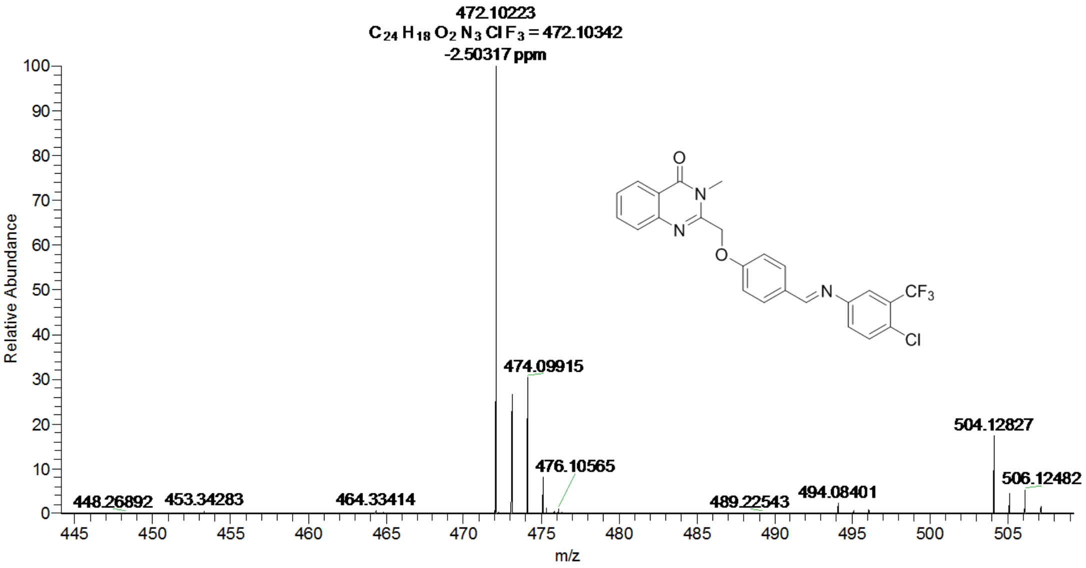

Fig.S48 HR-MS (ESI) spectrogram of compound 5p
